# Screening of heat-killed lactic acid bacteria based on inhibitory activity against oral bacteria and effects of oral administration of heat-killed *Ligilactobacillus salivarius* CP3365 on periodontal health in healthy participants: a double-blinded, randomized, placebo-controlled trial

**DOI:** 10.1080/20002297.2023.2250649

**Published:** 2023-08-28

**Authors:** Shinji Sakata, Yukiko Sakamaki, Masahiro Yuki, Tsutomu Sugaya, Tatsuhiko Hirota

**Affiliations:** a Core Technology Laboratories, Asahi Quality & Innovations, Ltd, Moriya-Shi, Ibaraki, Japan; b Periodontology & Endodontology Department of Oral Health Science Faculty of Dental Medicine, Hokkaido University, Kita-ku, Sapporo, Hokkaido, Japan

**Keywords:** Bleeding on probing (BOP), dental plaque, heat-killed lactic acid bacteria, *Ligilactobacillus salivarius* CP3365, probing pocket depth (PPD)

## Abstract

**Objectives:**

The aims of this study were to select heat-killed lactic acid bacteria (HKL) with antibiotic activity and investigate the efficacy of this bacteria in maintaining periodontal parameters in healthy participants.

**Materials and methods:**

An in vitro evaluation was conducted to assess the inhibitory efficacy of lactic acid bacteria against Porphyromonas gingivalis and Fusobacterium nucleatum subsp. nucleatum. The effects of HKL administration on various parameters (plaque control record, bleeding on probing, and probing pocket depth) were assessed in a randomized, placebo-controlled trial. Participants in the test and placebo groups (*n* = 32) consumed oral tablets containing placebo or HKL daily for 8 weeks. Oral bacteria in supra-plaque and saliva were identified using 16S rRNA gene community profiling analysis.

**Results:**

Heat-killed *Ligilactobacillus salivarius* CP3365 significantly (*p* < 0.05) decreased the viability of oral bacteria and was selected for clinical trials. Administration of HKL CP3365 significantly (*p* < 0.05) inhibited increases in each parameter. No changes in the relative abundance of P. gingivalis or F. nucleatum subsp. nucleatum were detected by HKL CP3365, but the relative abundance of oral bacteria (genera Porphyromonas, Fusobacterium, and Haemophilus) was significantly (*p* < 0.05) decreased.

**Conclusion:**

HKL CP3365 effectively inhibited oral bacteria growth and was useful for maintaining periodontal health.

**Clinical Trial Registration:**

[https://www.umin.ac.jp/ctr/index.htm], identifier [UMIN000045656].

## Introduction

Several hundred species of microorganisms exist in the oral environment [1], forming a microbial ecosystem surrounding the tooth, tongue, and buccal mucosa [2]. Certain microorganisms on teeth can form dental plaque, which is an aggregate made up of many types of bacteria and their metabolites [3,4]. Oral health is maintained by the balance between pathogenic dental plaque and host tissues [5]. However, when the balance is disrupted by factors such as changes in the growth of plaque [6] or changes in the host’s susceptibility to disease, such as obesity [7], smoking [8], hormonal imbalance due to pregnancy or menopause [9], or nutritional status [10], dental disease may manifest [5,10]. Therefore, it is important to control the deterioration of the host’s susceptibility to oral disease and manage dental plaque through oral hygiene.

Professional oral hygiene procedures are vital for maintaining oral health; however, daily self-care is also important for dental and gingival health [11]. Toothbrushes, dental floss, and chemical plaque control agents such as dentifrices and mouthwashes are important tools in oral self-care [12]. However, brushing alone removes only ~ 49.2% of plaque, regardless of the use of dentifrice [13]. Weijden et al. [14] showed that 1 min of brushing removes 39% of plaque; however, almost half of the volunteers in that study admitted that they did not brush for the full minute and that large amounts of plaque remained.

Other studies have found that electric toothbrushes are more effective at removing plaque than manual toothbrushes [15–17], but electric toothbrushes cannot be fully effective without professional oral hygiene instruction [16]. Chlorhexidine gluconate, triclosan, and cetylpyridinium chloride are commonly included as antibacterial agents in dentifrices and mouthwashes [18,19]. However, the Pharmaceutical Affairs Law of Japan set their maximum concentrations below their effective doses [11]. Hence, they may have limited efficacy in dental plaque control, and there is a need for alternative solutions.

Live lactic acid bacteria, formulated as probiotics, have demonstrated efficacy at normalizing the gut microbiome, and they have immunopotentiation activity [20,21]. Lactic acid bacteria have demonstrated activity against oral bacterial pathogens [22,23]. Living lactic acid bacterial strains such as *Limosilactobacillus* (*Lactobacillus*) *reuteri* [24,25] and *Ligilactobacillus* (*Lactobacillus*) *salivarius* (*L. salivarius*) [22,26] were effective against oral bacterial pathobionts in a patient with periodontal disease. In addition, the previous studies showed that administration of *L. reuteri* lozenges improved several periodontal health parameters, including bleeding on probing (BOP), gingival index, plaque control record (PCR), and probing pocket depth (PPD), in healthy participants [25]. Conversely, live lactic acid bacteria has been shown to cause dental caries [27,28]. The biofilm formation capacity of *Lactobacillus* spp. was enhanced in the presence of *Streptococcus mutans*. The presence of lactic acid bacteria on tooth surfaces increases the risk of caries by secreting lactic acid [29]. When lactic acid bacteria are administered as probiotics for oral health, it is essential to consider both their advantages and disadvantages in oral health. For these reasons, heat-killed lactic acid bacteria (HKL) showed great potential as a feasible option for oral administration because they secrete no lactic acid, contain no live bacteria, and function as postbiotics.

The present study selected and evaluated the efficacy of HKL as a postbiotic for the establishment and maintenance of dental and gingival health.

## Materials and methods

### Antibacterial activity test

#### Microorganisms and culture conditions

The bacterial strains used in the present study are shown in Table 1. Lactic acid bacteria were provided by the Japan Collection of Microorganisms (JCM; Ibaraki, Japan) and the Asahi Group Holdings (Ibaraki, Japan). The *Lactobacillus* strains were anaerobically cultured using an Anaero Pack system (Mitsubishi Gas Chemical Co., Tokyo, Japan) and in *Lactobacillus* Man – Rogosa–Sharpe (MRS) agar (Difco, Becton Dickinson, Sparks, MA, USA) at 37°C for 24 h. Colonies were suspended in 40 mL MRS broth (Difco, Becton Dickinson) and anaerobically incubated at 37°C for 24 h. The cells were collected by centrifugation at 2187 × *g* for 15 min and washed and suspended in distilled water (DW). The suspensions were autoclaved at 105°C for 30 min, and the cells were collected by freeze-drying to prepare the HKL.

**Table 1.** Strains of bacteria used in this study.

Bacteria	Species name	Strain number	Origin	Heat-killed bacterial cell weight (mg/mL)
Lactic acid bacteria	*Amylolactobacillus amylophilus*	JCM 1125^T^	RIKEN-BRC JCM	0.2
	*Furfurilactobacillus rossiae*	JCM 16,176^T^	RIKEN-BRC JCM	1.2
	*Lacticaseibacillus casei*	JCM 1134^T^	RIKEN-BRC JCM	1.3
	*Lacticaseibacillus paracasei* subsp. *paracasei*	JCM 8130^T^	RIKEN-BRC JCM	1.2
	*Lacticaseibacillus paracasei* subsp. *tolerans*	JCM 1171^T^	RIKEN-BRC JCM	0.8
	*Lacticaseibacillus rhamnosus*	JCM 1136^T^	RIKEN-BRC JCM	2.7
	*Lacticaseibacillus zeae*	JCM 11,302^T^	RIKEN-BRC JCM	1.9
	*Lactiplantibacillus plantarum*	JCM 1100	RIKEN-BRC JCM	1.8
	*Lactobacillus acidophilus*	JCM 1132^T^	RIKEN-BRC JCM	1.4
	*Lactobacillus amylovorus*	JCM 1126^T^	RIKEN-BRC JCM	2.1
	*Lactobacillus crispatus*	JCM 1185^T^	RIKEN-BRC JCM	0.9
	*Lactobacillus delbrueckii* subsp. *bulgaricus*	JCM 1002^T^	RIKEN-BRC JCM	0.9
	*Lactobacillus delbrueckii* subsp. *delbrueckii*	JCM 1012^T^	RIKEN-BRC JCM	1.3
	*Lactobacillus delbrueckii* subsp. *lactis*	JCM 1248^T^	RIKEN-BRC JCM	0.8
	*Lactobacillus gasseri*	JCM 1131^T^	RIKEN-BRC JCM	1.9
	*Lactobacillus helveticus*	JCM 1120^T^	RIKEN-BRC JCM	1.1
	*Latilactobacillus curvatus*	JCM 1096^T^	RIKEN-BRC JCM	0.9
	*Latilactobacillus sakei* subsp. *carnosus*	JCM 11,031^T^	RIKEN-BRC JCM	0.7
	*Lentilactobacillus farraginis*	JCM 14,108^T^	RIKEN-BRC JCM	1.2
	*Levilactobacillus brevis*	JCM 1059^T^	RIKEN-BRC JCM	0.5
	*Ligilactobacillus acidipiscis*	JCM 10,692^T^	RIKEN-BRC JCM	0.5
	*Ligilactobacillus salivarius*	CP3365	ASAHI	3.2
	*Ligilactobacillus salivarius*	JCM 1231^T^	RIKEN-BRC JCM	2.9
	*Ligilactobacillus salivarius*	JCM 1040	RIKEN-BRC JCM	1.3
	*Ligilactobacillus salivarius*	JCM 1150	RIKEN-BRC JCM	2.8
	*Ligilactobacillus salivarius*	JCM 7706	RIKEN-BRC JCM	2.6
	*Ligilactobacillus salivarius*	JCM 7712	RIKEN-BRC JCM	2.2
	*Limosilactobacillus fermentum*	JCM 1173^T^	RIKEN-BRC JCM	0.3
	*Limosilactobacillus mucosae*	JCM 12,515^T^	RIKEN-BRC JCM	1.4
	*Limosilactobacillus oris*	JCM 11,028^T^	RIKEN-BRC JCM	1.0
	*Limosilactobacillus reuteri* subsp. *reuteri*	JCM 1112^T^	RIKEN-BRC JCM	1.7
	*Liquorilactobacillus oeni*	JCM 18,036^T^	RIKEN-BRC JCM	1.7
	*Paucilactobacillus kaifaensis*	JCM 33,831^T^	RIKEN-BRC JCM	0.4
Oral bacteria	*Fusobacterium nucleatum* subsp. *nucleatum*	JCM 8532^T^	RIKEN-BRC JCM	-
	*Fusobacterium periodonticum*	JCM 12,991^T^	RIKEN-BRC JCM	-
	*Haemophilus parainfluenzae*	ATCC 33,392^T^	ATCC	-
	*Porphyromonas gingivalis*	ATCC 33,277^T^	ATCC	-
	*Porphyromonas pasteri*	JCM 30,531^T^	RIKEN-BRC JCM	-

Note: Bacterial cells were harvested from MRS broth and dried by freeze-drying.

*Porphyromonas gingivalis* (*P. gingivalis*) ATCC 33,277^T^ and *Haemophilus parainfluenzae* (*H. parainfluenzae*) ATCC 33,392^T^ were obtained from American Type Culture Collection (ATCC; Manassas, VA, USA). *Fusobacterium nucleatum* subsp. *nucleatum* (*F. nucleatum* subsp. *nucleatum*) JCM 8532^T^, *Porphyromonas pasteri* (*P. pasteri*) JCM 30,531^T^, and *Fusobacterium periodonticum* (*F. periodonticum*) JCM 12,991^T^ were provided by JCM. We cultured *P. gingivalis*, *F. nucleatum* subsp. *nucleatum*, and *F. periodonticum* in modified Gifu anaerobic medium (GAM broth; Nissui Pharmaceuticals, Tokyo, Japan) under anaerobic conditions at 37°C for 24 h. Colonies were inoculated in GAM broth (Nissui Pharmaceuticals) under anaerobic conditions at 37°C for 24 h. *Porphyromonas pasteri* was cultured in ABHK agar (Nissui Pharmaceuticals) under anaerobic conditions at 37°C for 48 h. A colony was inoculated in 3.7% (w/v) brain heart infusion (BHI; Nissui Pharmaceuticals) broth containing 5 μg/mL hemin (Nacalai Tesque, Kyoto, Japan), 0.5 μg/mL menadione (Nacalai Tesque), and 0.5% (w/v) yeast extract (Difco, Becton Dickinson) under anaerobic conditions at 37°C for 48 h. *Haemophilus parainfluenzae* was cultured in trypticase soy agar (Difco, Becton Dickinson) containing 5% (v/v) horse blood under aerobic conditions at 37°C for 24 h. Colonies were inoculated in BHI broth supplemented with 2 mg/mL nicotinamide adenine dinucleotide (Nacalai Tesque) under aerobic conditions at 37°C for 24 h.

## Inhibitory viability activity assay of *Lactobacillus* strains against oral bacteria

The microbial cell viability assay was performed based on antibacterial activity using a microbial viability assay kit (WST-8; Dojindo Laboratories, Kumamoto, Japan) according to the manufacturer’s instructions. The HKLs were suspended in 0.75% (w/v) NaCl to a density of 5 mg/mL. Then, 0.5 mL HKL suspension was inoculated into 4.5 mL bacterial pathogen suspension and adjusted to OD_660_ = 0.3. The mixture was incubated at 37°C for 30 min. Then, 20 μL WST-8 test solution was added to 180 μL incubated mixture in a 96-well plate, and the latter was incubated at 37°C. After 0 and 120 min, OD_460_ was measured with a MULTISKAN GO spectrophotometer (Thermo Fisher Scientific, Waltham, MA). Bacterial viability was determined from ΔOD_460_ (120–0 min). The assays were performed at least in triplicate.

## Effects of administration of heat-killed *Ligilactobacillus salivarius* CP3365 on periodontal health in healthy participants

### Study procedures

The design and protocol of the present study were approved by the Ethics Committees of Akanuma Hospital (Hokkaidou, Japan) (No. IBR 2106A; 17 March 2021), and the research was conducted according to the Declaration of Helsinki revised in Fortaleza, Brazil in 2013. The protocol was registered in the UMIN Clinical Trials Registry (No. UMIN000045656). All participants participating in the research understood the nature of the project and furnished prior written consent.

### Clinical trial design

The present study followed a randomized, double-blind, placebo-controlled design. The experimental period was 8 weeks between September and November 2021. The participants were assigned to a placebo group or a heat-killed *L. salivarius* CP3365 (HKL CP3365) group. From the day after the first clinical examination, the participants consumed either a tablet containing HKL CP3365 or a placebo tablet in which dextrin was substituted for the HKL CP3365. Each participant was instructed to lick, dissolve, and swallow the tablets after each meal (three times a day) without chewing them, in the same manner as a lozenge. Subjects were instructed to take one tablet at a time, for a total of three tablets. Clinical examinations were performed at 0, 4, and 8 weeks at Akanuma Hospital. During the study period, the participants were instructed to assess their health condition, maintain oral health care, and record their tooth-brushing frequency and type of toothbrush used. To evaluate their subjective symptoms, the participants maintained daily records of their test food ingestion and usage of all medication. The trial was managed by Kyowa Trial Co., Ltd. (Hokkaido, Japan).

### Participants

The study participants were males and females between 20 and 70 years of age. They were outwardly healthy and met the following criteria: 1) not currently visiting dentists for the treatment of cavities, gingivitis, or periodontitis; 2) ≥20 remaining teeth; 3) <10% of PPD over 4 mm and < 10% of BOP.

The exclusion criteria of the trial stage were as follows: 1) diagnosis of gingivitis, periodontitis, or dental caries at baseline; 2) medical history of diabetes, chronic kidney disease, gastrointestinal disorders, lung disease, cancers, or other serious disease, or consumption of medication for these conditions; 3) participation in a clinical trial requiring consumption of food (or medication) or application of cosmetics (or medication); 4) participation in a clinical trial within 4 weeks before the start of the present study; 5) removable dentures; 6) antibiotic consumption within 4 weeks of the onset of the present study; 7) food allergy, lactose intolerance, or illness in response to dairy product consumption; 8) pregnancy or lactation; 9) excessive alcohol and/or tobacco consumption; 10) objections to the principles and parameters of the study; and 11) an expert medical assessment of unsuitability for the present study for any other reasons.

The exclusion criteria of the analysis were as follows: 1) diagnosis of gingivitis or periodontitis at baseline; 2) consumption of < 90% of the test food; 3) continued or repeated consumption of health food supplements or medications that could affect the outcome of the present study; 4) lifestyle change such as excessive oral care; and 5) an expert medical assessment of unsuitability for the present study for any other reasons.

### Sample size

Sample sizes were determined in a preliminary study (data not shown). The mean PPD sizes for the CP3365 and placebo groups were 1.7 ± 0.2 mm and 2.1 ± 0.2 mm, respectively, by the end of the study. Thus, a required minimal sample size of 21 participants was determined with power = 90% and *p* < 0.05, according to G-power v. 1.9.1 (Franz Faul; University of Kiel, Kiel, Germany). Considering the expected rates of dropout and protocol violation, 64 participants were recruited for the present study.

### Outcomes

The parameters of PCR, BOP, and PPD were evaluated as the primary outcomes. The secondary outcome was the oral microbiota. Physical examinations and hematological and biological blood testing as well as height, weight, body mass index (BMI), body fat percentage, temperature, systolic and diastolic blood pressure, and pulse rate were measured for each study participant.

### Selection, randomization, and blinding

Sixty-four of the participants who provided informed consent were deemed eligible by a physician. They were randomly allocated either to the placebo group or the HKL CP3365 group (*n* = 32) by the stratified block randomization method based on participant sex, age, PCR, BOP, and PPD. Allocation and assignment were performed by an allocation controller (KM) using a computerized random number generator. The allocation controller locked the assignment tables until key opening day.

### Experimental tablets

All experimental tablets contained sorbitol, maltodextrin, fine silicon dioxide, calcium stearate, and hydroxypropyl cellulose. Cultured CP3365 for clinical study was sterilized at 95°C for 5 min. Each test tablet contained 4.4 × 10^9^ HKL CP3365 cells. Each placebo tablet contained additional crystalline cellulose.

### Clinical measurements

The PPD was measured with a plastic periodontal probe designed for dental implants (BSA Sakurai, Imabari, Ehime, Japan). The torque control was set to 0.25 N. Measurements were made at four sites per tooth. Any bleeding that appeared with 30 s of probing indicated a BOP-positive site. The full-mouth prevalence (%) of BOP was calculated. Dental plaque score was assessed based on the O’Leary, Dark & Naylor plaque control record [30]. Participants were requested to refrain from brushing their teeth one day before the clinical examination. All clinical measurements were conducted by two dental hygienists whose technical skill was deemed comparable prior to the onset of the study, and they also carried out oral examinations of randomly assigned examinees. Both were blinded to the group assignments of participants.

### Oral microbiota measurements

Unstimulated salivary samples (≥5 mL) were collected in sterile plastic tubes. Supragingival plaque samples were collected with sterilized swabs from the buccal surfaces of all molars. Each sample was stored at − 20°C until use. DNA was extracted from each sample with a DNeasy blood & tissue kit (Qiagen, Hilden Germany). The 16S rRNA gene amplicon sequence library was prepared according to the instructions of the kit manufacturer. DNA concentration was determined by NanoDrop (Thermo Fisher Scientific). The DNA samples were stored at − 20°C until use. The 16S rRNA gene library was compiled according to the manufacturer’s protocol (Illumina Library Preparation Guide, No. 15044223, Rev. B; Illumina, San Diego, CA, USA). The 16S rRNA gene (V3–V4 regions) was amplified via polymerase chain reaction (PCR) using the 341F and 806 R primers. The PCR amplicon was purified with AMpure XP (NIPPON Genetics, Tokyo, Japan). Sequencing was conducted using an Illumina MiSeq sequencing system (Illumina) and with an Illumina MiSeq reagent kit v.3 (600 cycles) (Illumina). QIIME2 (Quantitative Insights into Microbial Ecology; http://qiime2.org/) v. 2021.2 was used to analyze the sequences. Chimeric and noise sequences were removed, and the paired-end sequences were merged with the dada2 plug-in of QIIME2. Sequence taxonomy was determined by performing a BLAST search (https://blast.ncbi.nlm.nih.gov/Blast.cgi) against the 16S rRNA gene sequences (HOMD 16S rRNA Ref Seq v. 15.22) in the Human Oral Microbiome Database (https://homd.org). Nearest-neighbor species with ≥ 98% identity were selected as candidates for each representative amplicon sequences variant (ASV). The 16S rRNA gene community profiling analysis was performed by Genome-Lead Co. Ltd. (Takamatsu, Japan).

### Statistical analysis

After confirming normal distribution of data, in vitro test scores were analyzed using one-way analysis of variance (ANOVA) with Dunnett’s post hoc. In the clinical test, inter-group data were analyzed usingχ^2^ tests and Wilcoxon rank-sum statistical tests, and the number of changes in periodontal parameter were analyzed using analysis of covariance (ANCOVA). Intra-group data were analyzed using the Wilcoxon signed rank-sum test. Statistical analyses were performed in SPSS v. 23 (IBM Corp., Armonk, NY, USA).

## Results

### Antibacterial activity of HKL bacterial strains and dried bacterial cells against oral bacteria

**Figure 1.**
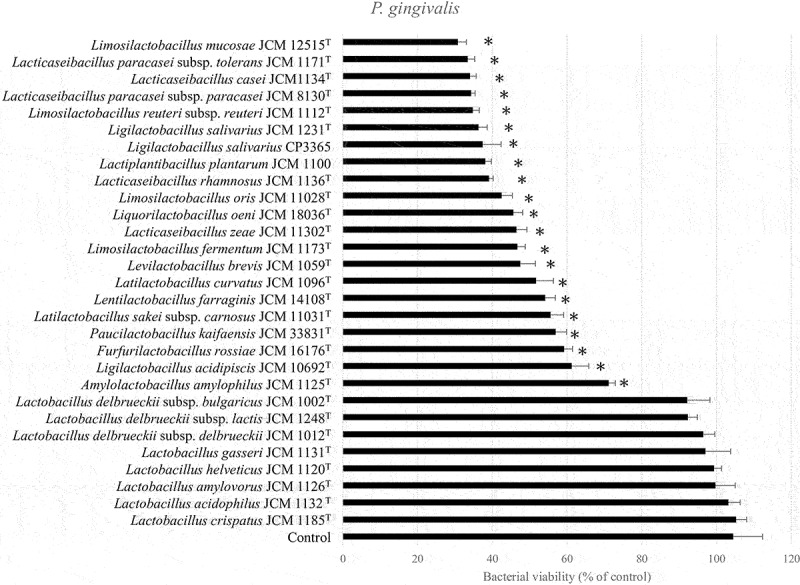
Bacterial viability measured using WST-8 assay. *Porphyromonas gingivalis* ATCC 33,277^T^ and the HKLs were co-inoculated into GAM broth and incubated at 37°C for 30 min. The OD_460_ was then measured at 0 and 120 min. Saline buffer was used as the control. Data are the means of three independent experiments. Significant differences were observed between the control and the HKLs. (*p < 0.05; one-way ANOVA with Dunnett’s post hoc test).

**Figure 2.**
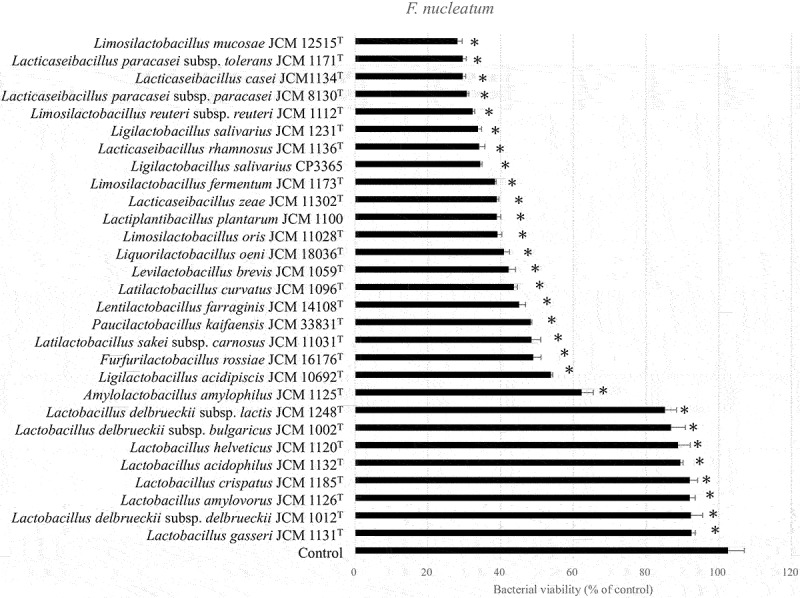
Bacterial viability measured using WST-8 assay. *Fusobacterium nucleatum* subsp. *nucleatum* JCM 8532^T^ and the HKLs were inoculated into GAM broth and incubated at 37°C for 30 min. The OD_460_ was then measured at 0 and 120 min. Saline buffer was used as the control. Data are the means of three independent experiments. Significant differences were observed between the control and the HKLs. (*p < 0.05; one-way ANOVA with Dunnett’s post hoc test).

**Figure 3.**
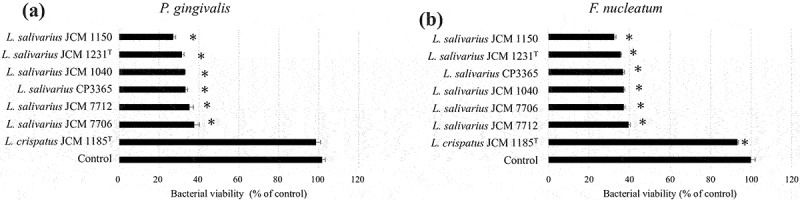
Bacterial viability measured using WST-8 assay. *Porphyromonas gingivalis* ATCC 33,277^T^ (a) and *Fusobacterium nucleatum* subsp. *nucleatum* JCM 8532^T^ (b), and the heat-killed *L Ligilactobacillus salivarius* strains, were inoculated into GAM broth and incubated at 37°C for 30 min. The OD_460_ was then measured at 0 and 120 min. Saline buffer was used as the control. Heat-killed *Lactobacillus crispatus* JCM 1185^T^ was the negative or weak positive control. Data are the means of three independent experiments. Significant differences were observed between the control and the HKLs. (*p < 0.05; one-way ANOVA with Dunnett’s post hoc test).

We evaluated the antibacterial ability of 28 lactic acid bacteria species from RIKEN-BRC JCM and our collections against oral bacteria. Bacterial viability of *P. gingivalis* ATCC 33,277^T^ was significantly (*p* < 0.05) decreased by 20 species outside the genus *Lactobacillus* (Figure 1). All strains decreased the viability of *F. nucleatum* subsp. *nucleatum* JCM 8532^T^. The genus *Lactobacillus* tended to have only weak antibacterial activity (Figure 2). Notably, cell weight of heat-killed *L. salivarius* was higher compared than that of the other species in MRS broth (Table 1). Hence, heat-killed *L. salivarius* was selected as the candidate for the clinical trial. To confirm the antibacterial activity of heat-killed *L. salivarius*, six strains, including their type strain, were evaluated. Antibacterial activity of heat-killed *Lactobacillus crispatus* JCM 1185^T^ against oral bacteria was used as a weak or negative control (Figure 3). All heat-killed *L. salivarius* strains significantly decreased the viability of *P. gingivalis* ATCC 33,277^T^ and *F. nucleatum* subsp. *nucleatum* JCM 8532^T^ (Figure 3). HKL CP3365 (dried bacterial cells) were obtained from MRS broth and had a density of 3.2 mg/mL (Table 1). The dried bacterial cells of other species were in the density range of 0.2–2.7 mg/mL. HKL CP3365 was selected as the strain for the subsequent clinical trials based on its antibacterial activity and its dried bacterial cell content.

### Effects of HKL CP3365 administration on periodontal parameters

#### Baseline characteristic

**Figure 4.**
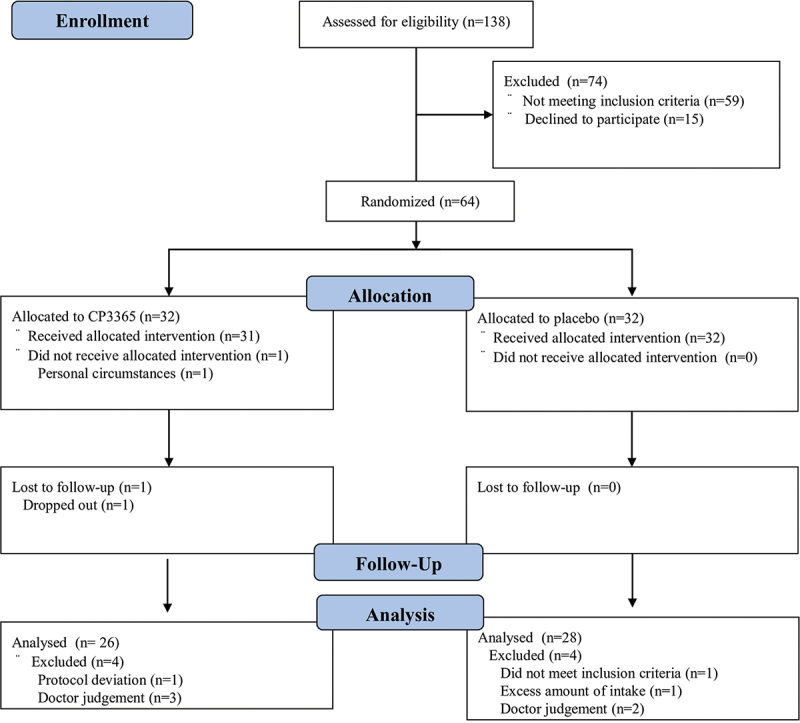
Consolidated standards of reporting trials (CONSORT) flow diagram.

An overview of the trial phases is shown in Figure 4. Sixty-four participants were selected and randomly allocated to the placebo group (*n* = 32) or the HKL CP3365 group (*n* = 32). Table 2 shows the baseline characteristics of the participants. Their parameters did not initially differ. One participant in the HKL CP3365 group withdrew from the trial after the first visit. Therefore, the full analysis set and safety analysis set reflect the fact that 32 participants in the placebo group and 31 participants in the HKL CP3365 group participated in the trial. One participant in the HKL CP3365 group dropped out during the test period, so 32 participants in the placebo group and 30 participants in the HKL CP3365 group completed it. Participants who met the exclusion criteria based on expert medical assessments were eliminated, leaving 28 in the placebo group and 26 in the HKL CP3365 group as per protocol set.

**Table 2.** Clinical characteristics of the study participants.

	Placebo	CP3365	*p*-value^b^
Parameters^a^	mean ± SE	mean ± SE	
Participants (*n*=)	32	32	
Gender (male:female)	14:18	15:17	0.62
Mean age	42.8 ± 11.5	45.6 ± 13.2	0.36
Clinical parameters			
Number of teeth	27.6 ± 1.7	27.5 ± 2.1	0.83
PCR (%)	57.6 ± 27.7	54.5 ± 26.6	0.58
BOP (%)	1.67 ± 2.42	1.59 ± 2.13	0.75
PPD (mm)	1.86 ± 0.26	1.87 ± 0.31	0.90
Brushing frequency (times per day)	2.0 ± 0.1	2.0 ± 0.2	0.97

Note: ^a^PCR: plaque control record, BOP: bleeding on probing, PDD: probing pocket depth. χ^b^ Score of gender was evaluated using the χ^2^ test, and the other parameters were evaluated using the Wilcoxon rank-sum test.

#### Changes in periodontal parameters

The results of the periodontal parameter measurements are shown in Table 3. In the placebo group, the PCR and BOP significantly increased after 4 weeks, and all parameters significantly increased after 8 weeks compared with those at 0 week. In the HKL CP3665 group, the BOP was temporarily increased at 4 weeks, and all parameters were not significantly changed at 8 weeks. All parameters had significantly increased in the placebo group at 8 weeks compared with those of the HKL CP3365 group.

**Table 3.** Differences in the periodontal parameters in this study.

		0 week				4 weeks					8 weeks				
Parameters^a^	Groups	Mean	±	SE	*p*-value^b^	Mean	±	SE	*p*-value^b^	*p*-value^c^	Mean	±	SE	*p*-value^b^	*p*-value^c^
PCR (%)	Placebo	62.0	±	4.8	0.59	66.1	±	4.9	0.19	<0.01	68.0	±	5.3	0.16	<0.01
	CP3365	61.7	±	4.4		59.7	±	3.9		0.99	61.1	±	4.4		0.72
BOP (%)	Placebo	1.70	±	0.35	0.83	4.06	±	0.83	0.22	<0.01	2.61	±	0.40	0.04	<0.01
	CP3365	1.61	±	0.33		2.47	±	0.36		0.02	1.46	±	0.29		0.75
PDD (mm)^c^	Placebo	1.85	±	0.05	0.94	1.89	±	0.05	0.58	0.07	1.95	±	0.05	0.16	<0.01
	CP3365	1.84	±	0.05		1.84	±	0.05		1.00	1.86	±	0.05		0.80
						4 weeks					8 weeks				
Number of changes in clinical parameters	Groups					Mean	±	SE		*p*-value^d^	mean	±	SE	*p*-value^d^	
ΔPCR (%)	Placebo					4.15	±	1.44		0.02	5.99	±	1.62	0.02	
	CP3365					−1.06	±	1.50			0.44	±	1.68		
ΔBOP (%)	Placebo					2.35	±	0.49		0.04	0.93	±	0.27	0.01	
	CP3365					0.88	±	0.51			−0.17	±	0.28		
ΔPDD (mm)	Placebo					0.04	±	0.02		0.23	0.10	±	0.03	0.03	
	CP3365					0.01	±	0.02			0.02	±	0.03		

Note: ^a^PCR: Plaque control record, BOP: Bleeding on probing, PPD, Probing pocket depth. ^b^The parameters between placebo and HKL CP3365 groups were evaluated using the Wilcoxon rank-sum test. ^c^The parameters between 0 and 4 or 8 weeks were evaluated using the Wilcoxon signed-rank test. ^d^ANCOVA with adjustment for each baseline value as covariates within the 4-week or 8-week period.

#### Oral microbiota analysis

Three of the 28 supragingival plaque samples, one of the 28 saliva samples in the placebo group, and one of the 26 supragingival plaque samples in the HKL CP3365 group were excluded because they had less than 2,000 reads and were therefore considered to be of low sequencing quality (Figure S1).

**Figure 5.**
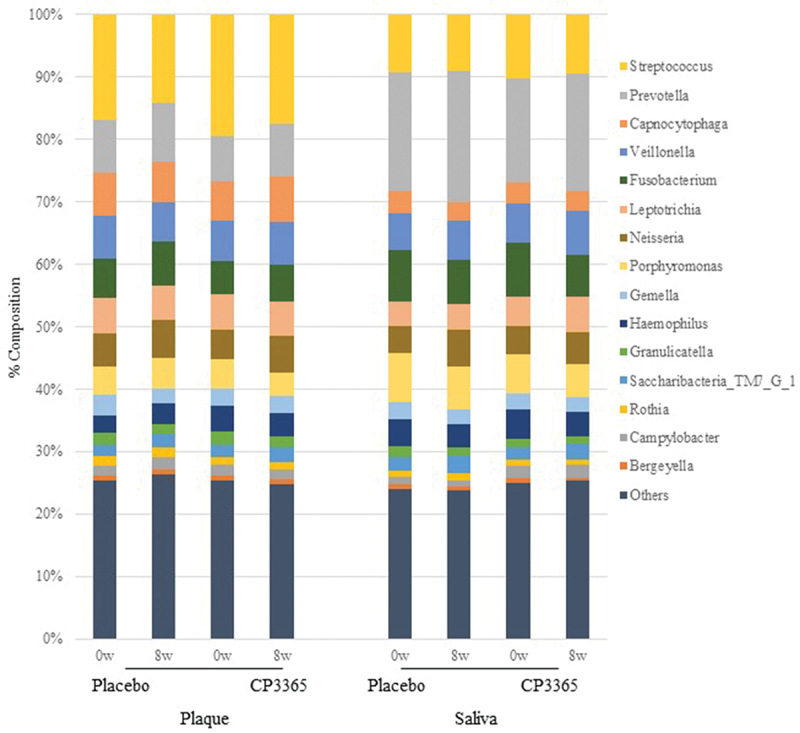
Approximate relative abundance of > 75% of bacterial genera identified in saliva and supragingival plaque of healthy participants determined by using 16S rRNA gene sequencing.

Takeshita et al. [31] reported that there are bacterial genera shared among individuals in the oral microbiome. They also defined ASVs detected in more than 75% of the subjects as highly common among individuals. In the present study, 15 bacterial genera out of 129 were detected in more than 75% of all examined individuals; therefore, these 15 genera were selected as the core oral bacterial genera in accordance with Takeshita et al. [31], and the remaining 114 were tabulated as other genera (Figure 5). Changes in the relative abundance in supra-plaque samples after 8 weeks were significantly decreased in genera *Neisseria* and *Porphyromonas* in the HKL CP3365 group compared with those in the placebo group (Table 4). For intra-group analysis, the relative abundance of genus *Porphyromonas* in the HKL CP3365 group was significantly decreased (Table 4). In the salivary microbiota, the abundances of genera *Fusobacterium* and *Haemophilus* in the HKL CP3365 group at 8 weeks were significantly decreased compared to those of the placebo group (Table 5). There were also some bacterial species that showed significant changes in their species-level comparisons: the supra-plaque samples of *Neisseria* sp, the saliva sample *F. periodonticum*, and *H. parainfluenzae*. Changes in *P. pasteri* in the supra-plaque samples were *p* = 0.06 (Table S1 and S2).

**Table 4.** Differences in the relative abundances of supragingival plaque microbiota at the genus level.

Taxonomic information	Groups	0 week				Detection rate (%)	8 weeks					Δ8 weeks (8 w-0 w)				Detection rate (%)
Genus level		Mean	±	SE	*p*-value^a^		Mean	±	SE	*p*-value^a^	*p*-value^b^	Mean	±	SE	*p*-value^a^	
*Bergeyella*	Placebo	0.93	±	0.19	0.48	84.0	0.86	±	0.16	0.82	0.61	−0.06	±	0.11	0.92	84.0
	CP3365	0.74	±	0.15		88.0	0.84	±	0.19	0.99		0.09	±	0.19		88.0
*Campylobacter*	Placebo	1.41	±	0.25	0.51	88.0	1.66	±	0.35	0.49	0.57	0.22	±	0.23	0.79	88.0
	CP3365	1.88	±	0.34		96.0	1.78	±	0.25	0.67		−0.09	±	0.30		96.0
*Capnocytophaga*	Placebo	7.91	±	0.99	0.55	100.0	6.97	±	0.81	0.28	0.29	−0.94	±	0.92	0.13	100.0
	CP3365	7.12	±	0.95		100.0	8.20	±	0.90	0.26		1.09	±	0.72		100.0
*Fusobacterium*	Placebo	7.59	±	0.65	0.53	100.0	6.05	±	0.52	0.01	0.31	−1.54	±	0.52	0.90	100.0
	CP3365	8.23	±	0.64		100.0	6.59	±	0.62	<0.01		−1.64	±	0.56		100.0
*Gemella*	Placebo	3.60	±	0.37	0.14	100.0	3.10	±	0.29	0.33	0.72	−0.49	±	0.42	0.33	100.0
	CP3365	2.89	±	0.27		100.0	3.09	±	0.30	0.88		0.19	±	0.35		100.0
*Granulicatella*	Placebo	2.39	±	0.24	0.06	100.0	2.55	±	0.31	0.90	0.19	0.16	±	0.34	0.52	100.0
	CP3365	1.83	±	0.24		100.0	2.08	±	0.31	0.30		0.25	±	0.28		100.0
*Haemophilus*	Placebo	3.23	±	0.44	0.38	96.0	4.04	±	0.77	0.67	0.85	0.78	±	0.79	0.66	96.0
	CP3365	3.79	±	0.51		96.0	3.66	±	0.43	0.74		−0.13	±	0.44		96.0
*Leptotrichia*	Placebo	6.14	±	0.70	0.88	100.0	6.25	±	0.62	0.99	0.95	0.11	±	0.73	0.46	100.0
	CP3365	5.74	±	0.59		100.0	6.48	±	0.70	0.25		0.74	±	0.63		100.0
*Neisseria*	Placebo	5.24	±	0.92	0.27	96.0	5.69	±	0.99	0.33	0.78	0.51	±	0.67	0.02	96.0
	CP3365	7.20	±	1.08		100.0	5.80	±	0.90	0.04		−1.40	±	0.84		100.0
*Porphyromonas*	Placebo	5.17	±	0.78	0.57	100.0	5.70	±	1.04	0.66	0.71	0.87	±	0.58	0.02	100.0
	CP3365	5.67	±	0.73		100.0	4.38	±	0.48	0.02		−1.29	±	0.50		100.0
*Prevotella*	Placebo	10.11	±	1.25	0.53	100.0	8.12	±	1.14	0.02	0.09	−1.99	±	0.88	0.23	100.0
	CP3365	10.94	±	1.15		100.0	10.24	±	1.00	0.38		−0.70	±	0.90		100.0
*Rothia*	Placebo	1.61	±	0.26	0.23	92.0	1.44	±	0.25	0.94	0.99	−0.15	±	0.30	0.99	92.0
	CP3365	1.36	±	0.33		80.0	1.44	±	0.29	0.88		0.06	±	0.22		80.0
*Saccharibacteria* TM7 G-1	Placebo	2.00	±	0.31	0.95	100.0	1.95	±	0.29	0.90	0.96	−0.06	±	0.35	0.85	100.0
	CP3365	2.38	±	0.45		100.0	2.37	±	0.48	0.74		−0.01	±	0.49		100.0
*Streptococcus*	Placebo	19.84	±	1.62	0.049	100.0	22.79	±	1.81	0.11	0.19	2.95	±	1.85	0.99	100.0
	CP3365	15.79	±	1.43		100.0	19.57	±	1.61	<0.01		3.78	±	0.95		100.0
*Tannerella*	Placebo	1.24	±	0.29	0.10	88.0	1.02	±	0.22	0.33	0.12	−0.19	±	0.19	0.73	88.0
	CP3365	1.85	±	0.31		76.0	1.64	±	0.29	0.66		−0.16	±	0.24		76.0
*Veillonella*	Placebo	8.16	±	0.61	0.40	100.0	7.65	±	0.80	0.18	0.79	−0.51	±	0.60	0.17	100.0
	CP3365	7.43	±	0.61		100.0	8.04	±	0.77	0.56		0.62	±	0.59		100.0

Note: ^a^The parameters between placebo and HKL CP3365 were evaluated using the Wilcoxon rank-sum test. ^b^The parameters between 0 and 8 weeks were evaluated using the Wilcoxon signed-rank test. ^c^The number of samples in each group was 25.

**Table 5.** Differences in the relative abundances of salivary microbiota at the genus level.

Taxonomic information		0 week				Detection rate (%)^c^	8 weeks					Detection rate (%)^c^	Δ8 weeks (8 w-0 w)			
Species level	Groups	Mean		SE	*p*-value^a^		Mean	±	SE	*p*-value^a^	*p*-value^b^		Mean	±	SE	*p*-value^a^
*Bergeyella*	Placebo	0.88	±	0.35	0.11	100.0	0.75	±	0.10	0.06	0.09	100.0	−0.13	±	0.29	0.16
	CP3365	0.54	±	0.11		100.0	0.51	±	0.09		0.71	100.0	−0.03	±	0.10	
*Campylobacter*	Placebo	1.13	±	0.09	0.41	100.0	2.12	±	0.17	0.80	<0.01	100.0	0.99	±	0.15	0.89
	CP3365	1.03	±	0.12		100.0	2.06	±	0.14		<0.01	100.0	1.04	±	0.15	
*Capnocytophaga*	Placebo	3.71	±	0.36	0.13	100.0	3.50	±	0.40	0.57	0.36	100.0	−0.21	±	0.44	0.23
	CP3365	2.96	±	0.38		100.0	3.14	±	0.39		0.62	100.0	0.18	±	0.24	
*Fusobacterium*	Placebo	8.93	±	0.75	0.15	100.0	9.11	±	0.73	0.04	0.74	100.0	0.19	±	0.62	0.40
	CP3365	7.38	±	0.74		100.0	6.82	±	0.69		0.62	100.0	−0.56	±	0.57	
*Gemella*	Placebo	2.98	±	0.19	0.02	100.0	2.76	±	0.25	0.35	0.68	100.0	−0.22	±	0.25	0.53
	CP3365	2.34	±	0.24		100.0	2.43	±	0.27		0.49	100.0	0.09	±	0.19	
*Granulicatella*	Placebo	1.77	±	0.15	0.22	100.0	1.25	±	0.11	0.43	<0.01	100.0	−0.52	±	0.15	0.66
	CP3365	1.48	±	0.13		100.0	1.18	±	0.08		0.02	100.0	−0.30	±	0.12	
*Haemophilus*	Placebo	4.40	±	0.37	0.11	100.0	5.08	±	0.31	0.02	0.049	100.0	0.68	±	0.37	0.34
	CP3365	3.79	±	0.26		100.0	4.02	±	0.29		0.40	100.0	0.22	±	0.27	
*Leptotrichia*	Placebo	4.16	±	0.40	0.68	100.0	5.05	±	0.56	0.32	0.13	100.0	0.89	±	0.49	0.51
	CP3365	4.34	±	0.44		100.0	5.91	±	0.64		0.03	100.0	1.57	±	0.59	
*Neisseria*	Placebo	4.35	±	0.42	0.17	100.0	4.69	±	0.36	0.56	0.27	100.0	0.34	±	0.31	0.16
	CP3365	6.03	±	0.80		100.0	5.23	±	0.43		0.34	100.0	−0.80	±	0.67	
*Porphyromonas*	Placebo	8.17	±	0.57	0.17	100.0	6.40	±	0.44	0.23	<0.01	100.0	−1.77	±	0.50	0.64
	CP3365	7.22	±	0.49		100.0	5.66	±	0.42		<0.01	100.0	−1.56	±	0.44	
*Prevotella*	Placebo	19.57	±	1.49	0.14	100.0	16.88	±	1.00	0.05	<0.01	100.0	−2.69	±	0.90	1.00
	CP3365	21.95	±	1.32		100.0	19.47	±	0.76		0.01	100.0	−2.48	±	1.04	
*Rothia*	Placebo	1.01	±	0.15	0.69	100.0	1.06	±	0.18	0.71	0.47	100.0	0.06	±	0.13	0.20
	CP3365	1.05	±	0.14		100.0	0.93	±	0.15		0.41	100.0	−0.12	±	0.12	
*Saccharibacteria* TM7 G-1	Placebo	2.31	±	0.25	0.25	100.0	2.05	±	0.20	0.34	0.68	100.0	−0.27	±	0.24	0.33
	CP3365	2.85	±	0.35		100.0	2.62	±	0.35		0.11	100.0	−0.23	±	0.30	
*Streptococcus*	Placebo	9.44	±	0.54	0.82	100.0	10.64	±	0.44	0.28	0.03	100.0	1.21	±	0.54	0.29
	CP3365	9.30	±	0.49		100.0	9.88	±	0.55		0.41	100.0	0.58	±	0.57	
*Tannerella*	Placebo	0.15	±	0.03	0.88	77.8	0.21	±	0.10	0.76	0.50	77.8	0.05	±	0.08	0.62
	CP3365	0.15	±	0.03		80.8	0.08	±	0.02		0.16	80.8	−0.06	±	0.03	
*Veillonella*	Placebo	6.03	±	0.31	0.13	100.0	6.62	±	0.41	0.21	0.16	100.0	0.60	±	0.42	0.62
	CP3365	6.54	±	0.25		100.0	7.52	±	0.37		0.01	100.0	0.98	±	0.36	

Note: ^a^The parameters between placebo and HKL CP3365 were evaluated using the Wilcoxon rank-sum test. ^b^The parameters between 0 and 8 weeks were evaluated using the Wilcoxon signed-rank test. ^c^The number of samples in the placebo group was 27, and that in the HKL CP3365 group was 26.

#### Antibacterial activity of heat-killed lactic acid bacterial strains against oral bacteria

**Figure 6.**
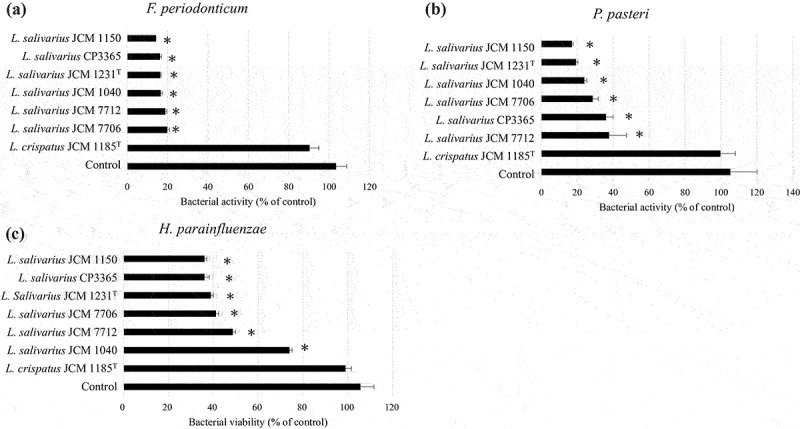
Bacterial viability measured using WST-8 assay. *Fusobacterium periodonticum* JCM 12,991^T^ (a), *Porphyromonas pasteri* JCM 30,531^T^ (b), *Haemophilus parainfluenzae* ATCC33392^T^ (c), and heat-killed *Ligilactobacillus salivarius* strains were inoculated into GAM broth and incubated at 37°C for 30 min. The OD_460_ was then measured at 0 and 120 min. Saline buffer was used as the control. Data are the means of three independent experiments. Significant differences were observed between the control and the HKLs. (*p < 0.05; one-way ANOVA with Dunnett’s post hoc test).

We tested whether HKL CP3365 had antimicrobial activity against four genera. First, we estimated the species-level composition of the four genera in which changes were identified (Table S1 and S2), and then, we estimated the major bacterial species comprising the genus based on the relative abundance and the amount of change. From these results, four species were selected as representatives (*F. periodonticum*, *H. parainfluenzae, Neisseria* sp, and *P. pasteri*) because they had a high relative abundance and the changes were significant or showed a significant trend. Of these, the unidentified strain *Neisseria* sp. were excluded from the study because the strains were not available, and *H. parainfluenzae, F. periodonticum*, and *P. pasteri* were then selected and examined for their antimicrobial activity. *Ligilactobacillus salivarius*, including HKL CP3365, significantly (*p* < 0.05) decreased the viability of all three oral bacterial pathogen species (Figure 6).

#### *Effects of HKL CP3365 on periodontal parameters in the presence or absence of* P. gingivalis

**Figure 7.**
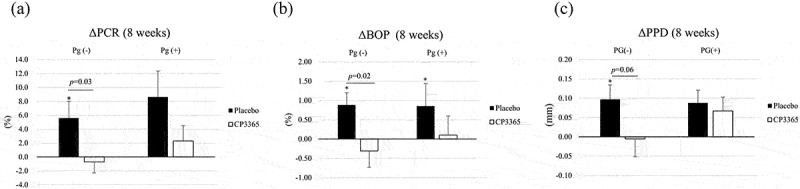
Stratified analysis of the periodontal parameters in the presence of *Porphyromonas gingivalis*. the numbers for participants were based on the existence of *P. gingivalis* in samples at 8 weeks (table S2). (a) PCR: plaque control record; (b) BOP: bleeding on probing; (c) PPD: probing pocket depth. *p*-values (inter-group) were determined using ANCOVA with adjustment for each baseline value. *: *p* < 0.05; Δ8 weeks (8 weeks–0 week) were evaluated using the Wilcoxon signed-rank test.

There was no change in the relative abundance of *P. gingivalis* after HKL CP3365 administration (Tables S1 and S2). Therefore, the relationship between *P. gingivalis* and the parameters was investigated. Participants were stratified based on the results of the detection (or no detection) of *P. gingivalis* in saliva (Table 2S). The results confirmed that the parameters (PCR and BOP) were exacerbated even in the absence of *P. gingivalis* (Figure 7). HKL CP3365 also inhibited these exacerbations.

#### Safety of HKL CP3365 administration

HKL CP3365 administration caused no clinically significant adverse events in the present study (data not shown).

## Discussion

The purpose of this study was to confirm whether HKL was useful in maintaining periodontal health. We selected strain HKL CP3365 among 28 species based on its antibacterial activity against *P. gingivalis* and *F. nucleatum* ssp. *nucleatum* (Figures 1, 2). We examined the effects of HKL CP3365 administration on various periodontal parameters. Among healthy participants in the placebo group, PCR and BOP progressively deteriorated over ~4 weeks. Conversely, HKL CP3365 maintained these parameters at healthy levels for >8 weeks (Table 3). Furthermore, an oral microbiota analysis showed that HKL CP3365 had no apparent effect on *P. gingivalis*, but it did exhibit an influence on the relative abundance of the genera *Fusobacterium, Haemophilus, Neisseria*, and *Porphyromonas* (Table 4).

To assess HKL CP3365 of antimicrobial activity, we then selected *F. periodonticum*, *H. parainfluenzae*, and *P. pasteri* for further study, excluding *Neisseria* sp., for which strains were not available. In vitro tests showed the antibacterial activity of HKL CP3365 for those three species (Figure 6). These results indicate that the proliferation of these bacteria was related to the maintenance of periodontal health. From these results, HKL CP3365 was found to inhibit the increase in dental plaque by suppressing plaque-forming bacteria, and it also inhibited the exacerbation of BOP and PPD. Those results showed that administration of HKL, as well as live lactic acid bacteria, had important periodontal health maintenance functions.

In the placebo group, significant increases in PCR, BOP, and PPD were observed at 8 weeks. Periodontal health has been shown to be closely related to oral hygiene [6,32,33]. Therefore, considering that the participants in the study were healthy participants without periodontal disease, changes in these periodontitis parameters could be related to the frequency of tooth brushing [34], tooth-brushing technique [14], and type of antibacterial agent [35,36]. At the start of the study, participants had the habit of brushing their teeth on average twice a day (Table 2), and we instructed them to maintain this frequency throughout the study period. Lang et al. [34] conducted a study on tooth-brushing frequency and periodontal health and reported that participants who brushed twice a day during the 6-week study period maintained healthy gingiva. Thus, brushing teeth twice a day is considered a satisfactory prerequisite for maintaining good periodontal health. However, in this study, the placebo group showed deteriorated periodontal health, suggesting that the deterioration in periodontal health was related to the quality (brushing technique and type of toothpaste) rather than the frequency of toothbrushing.

The PCRs in participants at 0 week ranged between 61.0% and 61.7% (Table 3). Previous studies have reported PCRs of 35%–46.3% [37,38] in healthy participants. Compared to these reports [37,38], the plaque scores of the participants in this study were relatively high compared to normal healthy participants. The PCRs could be considered as an indicator of brushing technique [39], with lower-quality brushing technique tending to result in more plaques. Therefore, it was considered that the brushing technique of the present participants was inadequate. In addition, as commercial toothpastes contain antimicrobial agents against oral bacteria [11], a toothpaste free of antimicrobial agents was specified in this study, and participants were instructed to use it during the study period. The oral effectiveness of antimicrobial agents had already been reported in several studies [40–42], and their use had been shown to be effective in supporting immature toothbrushing [41]. It was therefore considered that the use of toothpaste without antimicrobials for immature toothbrushing techniques could lead to deterioration of the periodontal health. Owing to these factors, it was considered that periodontal health was not maintained in the placebo group.

For these reasons, both groups had the same oral hygiene status, but the periodontal health status (BOP and PPD) of the HKL CP3365 group was maintained without deterioration during the study period. Considering the deterioration gingival health in the placebo group, the results suggested that the administration of HKL CP3365 reduced plaque accumulation owing to inadequate brushing and that it maintained periodontal health.

Although *P. gingivalis* is recognized as an important pathogen of oral diseases [43], the abundance of this bacterium does not necessarily directly indicate the risk of periodontitis [44], and host susceptibility and other bacterial composition may also affect the risk of developing periodontal disease [43]. In the present study, HKL CP3365 was selected for its antibacterial activity against *P. gingivalis* (Figure 1), and its ingestion may affect the relative abundance of *P. gingivalis* in the oral cavity; however, no change was observed in the 16S rRNA gene community profiling analysis (Table S1, S2). In addition, we performed quantitative measurements of *P. gingivalis* using quantitative real time polymerase chain reaction (q-PCR); no change was observed in the relative abundance due to the administration of HKL CP3365 (data not shown). No changes in host susceptibility [e.g. obesity [7], smoking [8], and other factors [9,10]] were observed, suggesting that the deterioration of these parameters may be related to changes in oral bacteria other than *P. gingivalis*. Therefore, we compared bacterial species composition at the genus level and found that the relative abundance of *Fusobacterium*, *Haemophilus*, *Neisseria*, and *Porphyromonas* in the participant’s oral cavity changed after administration of HKL CP3365. 16S rRNA gene community profiling analysis results suggest that the main species that constitute these genera include *F. periodonticum*, *H. parainfluenzae*, *Neisseria* sp., and *P. pasteri*, based on their relative abundances, and we hypothesized that their composition is related to the deterioration of periodontal health. Note that the change in *P. pasteri* was not significant but was selected because of its high abundance in the genus *Porphyromonas.*

*Haemophilus parainfluenzae* was previously detected in early oral biofilm formation [45]. It produced an adhesin that allowed it to co-aggregate with streptococci and adhere to the pellicle of the tooth surface [46]. Hence, *H. parainfluenzae* is a vital initiator of cell-cell interactions in early oral biofilm formation [46]. Furthermore, *P. pasteri* abundance is increased in mid-stage oral biofilm formation [45]. *Porphyromonas pasteri* was named by Sakamoto et al. [47] in 2015, and it had previously been named *Porphyromonas* sp. OT-279. Although its function in periodontal health was unclear, it is considered as a connecting link between the early and late stages of oral biofilm formation [45,48]. *Fusobacterium periodonticum* has been detected in late oral biofilm formation [31], and its *FadA* encodes an adhesin that resembles that of *Fusobacterium nucleatum* subsp. *nucleatum* and is associated with oral plaque development [49]. The present study showed that the three species were detected in nearly all participants and that their relative abundances were high. Therefore, we hypothesized that these oral bacteria contribute to the increase in oral plaque. HKL CP3365 inhibited the activity of each genus, including these three main bacterial species. This suggests that HKL CP3365 May directly prevent the development of oral plaque by inhibiting the activity of bacteria involved in plaque formation.

HKL CP3365 showed antimicrobial activity against *F. nucleatum subsp. nucleatum* in in vitro studies (Figure 2), but *F. nucleatum* subsp. *nucleatum* was not detected in clinical studies by 16S rRNA gene community profiling analysis (Table S1, S2). *Fusobacterium nucleatum* subsp. *nucleatum* is universally detected in the human oral cavity and was detected by species-specific PCR in more than 80% of Japanese participants [50]. However, in the present study, *F. nucleatum* subsp. *nucleatum* was not detected in supragingival plaque or saliva by 16S rRNA gene community profiling analysis. The reason for this was likely a technical problem between qualitative and quantitative analysis. The qualitative problem may be that *Fusobacterium* was not sufficiently detected at the species level. It has been reported that the 16S rRNA genes among *Fusobacterium* species are highly similar [51], and the 16S rRNA genes of *F. necrophorum*, *F. nucleatum* subsp. *animalis*, *F. nucleatum* subsp. *nucleatum*, and *F. nucleatum* subsp. *vinculum* detected in the present study are all highly similar to those of other bacteria. In the HOMD database [52], sequences showing more than 98.5% similarity are defined as species. The 16S rRNA gene community profiling analysis may not have had sufficient discriminatory power for these bacterial species. In addition, the number of sequence reads may have been insufficient as a quantitative problem. A previous study analyzed the oral flora using the same V3-V4 primers used in the present study and detected *F. nucleatum* subsp. *nucleatum* in 70% of healthy participants [53]; however, average number of reads [53] was approximately 10 times higher than that in our study. This suggests that the conditions of the bacterial flora analysis method in this study may not be suitable for the detection of *F. nucleatum*. Future studies should be conducted to understand the behavior of *Fusobacterium* spp. in the oral cavity using a more discriminating and sensitive method.

In this study, V3–4 was used because it was consistent with the Illumina protocol. Previously, V1–2, V1–3, V3–4, and V4–5 have been used to analyze oral bacteria [54–57]. Verma et al. [57] reported that 16S rRNA gene community profiling analysis analyses based on the oral microbiome mainly rely on primers in the V1–2 or V3–4 regions. Fragments of these 16S rRNA gene regions provide an adequate comprehensive representation of the bacterial phylum present in the given ecological environment. Teng et al. [56] also reported that V3–4 and V4–5 were more reproducible for bacterial phylum analysis than V1–3. Therefore, the analysis of oral bacteria by V3–4 was considered to have a certain degree of reliability. In other studies, V1–2 has been shown to be more suitable for species identification than were V3–4 [55] or V4–5 [54]. In the future, it will be necessary to select primers according to purpose.

Living lactic acid bacteria were effective against the microbial pathogens that cause periodontal disease. Lactic acid [58] and bacteriocins [59,60] produced by lactic acid bacteria acted directly on microbial pathogens in the oral cavity. Active components were present in the culture medium of lactic acid bacteria and has been studied as postbiotics [61]. In contrast, the HKL containing CP3365 did not contain extracellular components, as they had been washed. Although the definite active component of HKL CP3365 is currently unknown, Sulijaya et al. [62] found that 10-oxo-trans-11-octadecanoic acid from *L. plantarum* was effective against *P. gingivalis*. It was therefore expected that the active ingredient in the present study was not an exocrine secretion of the bacteria, but a bacterial component. The identification of the active ingredient will be a subject for further study.

The present study also confirmed that HKL, excluding *Lactobacillus* spp. as re-organized by Zheng et al. [63], had strong activity against *P. gingivalis*. While in a previous study [64] HKL from species not included in the genus *Lactobacillus* (*L. salivarius* subsp. *salicinius*, *L. reuteri*, *L. rhamnosus*, and *L. paracasei*) inhibited *P. gingivalis*, *Lactobacillus helveticus* and *L. acidophilus* did not. Therefore, it was considered possible that lactic acid bacteria (excluding *Lactobacillus* spp.) may have antimicrobial cellular components in common with HKL.

The mechanism of the action of HKL CP3365 May be its antibacterial activity against oral bacteria, and it was assumed that gingival health would be maintained if HKL CP3365 remained in the oral cavity for a certain time at a certain concentration. The test food was designed so that when dissolved at a typical salivary secretion rate (salivary secretion rate during stimulation: 10 mL/10 min), the concentration of lactic acid bacteria in saliva would be approximately the same as in the in vitro test (approximately 0.9 × 10^9^/mL). Although we did not instruct the subjects how long to lick and swallow the tablets, our tablets were manufactured with a hardness that allows them to dissolve in approximately 4–5 min, similar to the dissolving time of common tablet products in Japan, and the concentration of HKL CP3365 in the oral cavity was maintained at a concentration expected to have antimicrobial activity for approximately 12–15 min. Furthermore, preliminary comparison of the persistence of HKL CP3365 in saliva after ingestion showed that HKL CP3365 remained at 1/10–1/100 of the dose for 15–30 min (data not shown). This suggests that the concentration of HKL CP3365 was maintained to some extent in the oral cavity in the present study, indicating the oral antimicrobial action of HKL CP3365 against oral bacteria was effective and HKL CP3365 maintained gingival health. However, possible secondary actions of lactic acid bacteria, such as anti-inflammatory and immunostimulating actions, should be considered and investigated.

Since follow-up after lactobacilli intake was not conducted in this study, we did not investigate the persistence of lactobacilli after the intake of lactobacilli was terminated. It has been reported that the effect of lactobacilli intake is persistent even after discontinuation [65]. Therefore, we plan to study the possibility that changes in the bacterial flora caused by HKL CP3365 are maintained even after the intake of HKL CP3365 is terminated, thereby maintaining periodontal health. In addition, obesity [7], smoking [8], hormonal imbalances due to pregnancy and menopause [9], and nutritional balance [10] have been implicated as factors in gingivitis susceptibility, and it is necessary to investigate the efficacy against these in the future.

There were two major limitations to this study. First, the participants’ gingival condition was set at the same level as at screening, but it was not known whether the subsequent gingival condition of the examinees would improve or deteriorate. Second, we selected participants who had no periodontal disease as participants, so further study is needed to determine if HKL CP3365 can improve poor periodontal health.

Our results indicate that HKL CP3365 directly prevents dental plaque development by inhibiting the activity of oral bacteria to maintain periodontal health related to dental plaque and gingiva. We found that daily HKL CP3365 administration in healthy participants without periodontitis enhanced the effectiveness of their periodontal health care and maintained their periodontal health.

## Supplementary Material

Supplemental Material

## Data Availability

The sequencing data presented in the study are deposited in https://www.ncbi.nlm.nih.gov/. DRR426262-DRR426509.

## References

[1] Arweiler NB, Netuschil L. The oral microbiota. Adv Exp Med Bio. 2016;902:45–16. doi: 10.1007/978-3-319-31248-4_4 27161350

[2] Sharma N, Bhatia S, Sodhi AS, et al. Oral microbiome and health. AIMS Microbiol. 2018;4(1):42. doi: 10.3934/microbiol.2018.1.42 31294203 PMC6605021

[3] Kleinberg I. A mixed-bacteria ecological approach to understanding the role of the oral bacteria in dental caries causation: an alternative to Streptococcus mutans and the specific-plaque hypothesis. Crit Rev Oral Biol Med. 2002;13(2):108–125. doi: 10.1177/154411130201300202 12097354

[4] Mark Welch JL, Rossetti BJ, Rieken CW, et al. Biogeography of a human oral microbiome at the micron scale. Proc Natl Acad Sci, USA. 2016;113(6):E791–800. doi: 10.1073/pnas.1522149113 26811460 PMC4760785

[5] Kilian M, Chapple I, Hannig M, et al. The oral microbiome–an update for oral healthcare professionals. Br Dent J. 2016;221(10):657–666. doi: 10.1038/sj.bdj.2016.865 27857087

[6] Bamashmous S, Kotsakis GA, Kerns KA, et al. Human variation in gingival inflammation. Proc Natl Acad Sci, USA. 2021;118(27):e2012578118. doi: 10.1073/pnas.2012578118 34193520 PMC8271746

[7] Hegde S, Chatterjee E, Rajesh KS, et al. Obesity and its association with chronic periodontitis: a cross-sectional study. J Educ Health Promot. 2019;8:222. doi: 10.4103/jehp.jehp_40_19 31867386 PMC6905353

[8] Rivera‐Hidalgo F. Smoking and periodontal disease: a review of the literature. J Periodontol. 1986;57(10):617–624. doi: 10.1902/jop.1986.57.10.617 3534210

[9] Markou E, Eleana B, Lazaros T, et al. The influence of sex steroid hormones on gingiva of women. Open Dent J. 2009;3(1):114. doi: 10.2174/1874210600903010114 19812718 PMC2758498

[10] Jenzsch A, Eick S, Rassoul F, et al. Nutritional intervention in patients with periodontal disease: clinical, immunological and microbiological variables during 12 months. Br J Nutr. 2008;101(6):879–885. doi: 10.1017/S0007114508047776 18713481

[11] JSP Clinical Practice Guideline for the Periodontal Treatment [Internet]. Japan: The Japanese Society of Periodontology; 2015 [updated December 15; cited December 01]. Available from: https://www.perio.jp/publication/upload_file/guideline_perio_plan2015_en.pdf.

[12] Chapple IL, Van der Weijden F, Doerfer C, et al. Primary prevention of periodontitis: managing gingivitis. J Clin Periodontol. 2015;42:S71–S76. doi: 10.1111/jcpe.12366 25639826

[13] Valkenburg C, Slot DE, Bakker EW, et al. Does dentifrice use help to remove plaque? A systematic review. J Clin Periodontol. 2016;43(12):1050–1058. doi: 10.1111/jcpe.12615 27513809

[14] Van der Weijden GA, Hioe K. A systematic review of the effectiveness of self‐performed mechanical plaque removal in adults with gingivitis using a manual toothbrush. J Clin Periodontol. 2005;32(s6):214–228. doi: 10.1111/j.1600-051X.2005.00795.x 16128840

[15] Slot DE, Valkenburg C, Van der Weijden GA. Mechanical plaque removal of periodontal maintenance patients: a systematic review and network meta‐analysis. J Clin Periodontol. 2020;47(S22):107–124. doi: 10.1111/jcpe.13275 32716118

[16] Van der Weijden GA, Danser MM, Nijboer A, et al. The plaque‐removing efficacy of an oscillating/rotating toothbrush: a short‐term study. J Clin Periodontol. 1993;20(4):273–278. doi: 10.1111/j.1600-051X.1993.tb00357.x 8473538

[17] van der Weijden FA, Timmerman MF, Piscaer M, et al. A comparison of the efficacy of a novel electric toothbrush and a manual toothbrush in the treatment of gingivitis. Am J Dent. 1998 Sep;11(Spec No):S23–S28. 10530096

[18] Brookes ZL, Bescos R, Belfield LA, et al. Current uses of chlorhexidine for management of oral disease: a narrative review. J Dent. 2020;103:103497. doi: 10.1016/j.jdent.2020.103497 33075450 PMC7567658

[19] Rajendiran M, Trivedi HM, Chen D, et al. Recent development of active ingredients in mouthwashes and toothpastes for periodontal diseases. Molecules. 2021;26(7):2001. doi: 10.3390/molecules26072001 33916013 PMC8037529

[20] Isolauri E, Sütas Y, Kankaanpää P, et al. Probiotics: effects on immunity. Am J Clin Nutr. 2001;73(2):444s–450s. doi: 10.1093/ajcn/73.2.444s 11157355

[21] Van Baarlen P, Wells JM, Kleerebezem M. Regulation of intestinal homeostasis and immunity with probiotic lactobacilli. Trends Immunol. 2013;34(5):208–215. doi: 10.1016/j.it.2013.01.005 23485516

[22] Ishikawa H, Aiba Y, Nakanishi M, et al. Suppression of periodontal pathogenic bacteria in the saliva of humans by the administration of Lactobacillus salivarius TI 2711. Nihon Shishubyo Gakkai Kaishi (Journal Of The Japanese Society Of Periodontology). 2003;45(1):105–112. doi: 10.2329/perio.45.105

[23] Kang M, Oh J, Lee H, et al. Inhibitory effect of Lactobacillus reuteri on periodontopathic and cariogenic bacteria. J Microbiol. 2011;49(2):193–199. doi: 10.1007/s12275-011-0252-9 21538238

[24] Iniesta M, Herrera D, Montero E, et al. Probiotic effects of orally administered Lactobacillus reuteri‐containing tablets on the subgingival and salivary microbiota in patients with gingivitis. A randomized clinical trial. J Clin Periodontol. 2012;39(8):736–744. doi: 10.1111/j.1600-051X.2012.01914.x 22694350

[25] Schlagenhauf U, Rehder J, Gelbrich G, et al. Consumption of Lactobacillus reuteri‐containing lozenges improves periodontal health in navy sailors at sea: A randomized controlled trial. J Periodontol. 2020;91(10):1328–1338. doi: 10.1002/JPER.19-0393 32017092

[26] Shimauchi H, Mayanagi G, Nakaya S, et al. Improvement of periodontal condition by probiotics with Lactobacillus salivarius WB21: a randomized, double‐blind, placebo‐controlled study. J Clin Periodontol. 2008;35(10):897–905. doi: 10.1111/j.1600-051X.2008.01306.x 18727656

[27] Lapirattanakul J, Nomura R, Okawa R, et al. Oral lactobacilli related to caries status of children with primary dentition. Caries Res. 2020;54(2):194–204. doi: 10.1159/000506468 32235114

[28] Shimada A, Noda M, Matoba Y, et al. Oral lactic acid bacteria related to the occurrence and/or progression of dental caries in Japanese preschool children. Biosci Microbiota Food Health. 2015;34(2):29–36. doi: 10.12938/bmfh.2014-015 25918670 PMC4405395

[29] Yli‐Knuuttila H, Snäll J, Kari K, et al. Colonization of Lactobacillus rhamnosus GG in the oral cavity. Oral Microbiol Immunol. 2006;21(2):129–131. doi: 10.1111/j.1399-302X.2006.00258.x 16476023

[30] O’leary TJ, Drake RB, Naylor JE. The plaque control record. J Periodontol. 1972;43(1):38–42. doi: 10.1902/jop.1972.43.1.38 4500182

[31] Takeshita T, Kageyama S, Furuta M, et al. Bacterial diversity in saliva and oral health-related conditions: the hisayama study. Sci Rep. 2016;6(1):1–11. doi: 10.1038/srep22164 26907866 PMC4764907

[32] Löe H, Theilade E, Jensen SB. Experimental gingivitis in man. J Periodontol. 1965;36(3):177–187. doi: 10.1902/jop.1965.36.3.177 14296927

[33] Theilade E, Wright WH, Jensen SB, et al. Experimental gingivitis in man: II. A longitudinal clinical and bacteriological investigation. J Periodont Res. 1966;1(1):1–13. doi: 10.1111/j.1600-0765.1966.tb01842.x 4224181

[34] Lang NP, Cumming BR, Löe H. Toothbrushing frequency as it relates to plaque development and gingival health. J Periodontol. 1973;44(7):396–405. doi: 10.1902/jop.1973.44.7.396 4514570

[35] Brecx M, Netuschil L, Reichert B, et al. Efficacy of listerine®, meridol® and chlorhexidine mouthrinses on plaque, gingivitis and plaque bacteria vitality. J Clin Periodontol. 1990;17(5):292–297. doi: 10.1111/j.1600-051X.1990.tb01092.x 2355095

[36] Lorenz K, Bruhn G, Heumann C, et al. Effect of two new chlorhexidine mouthrinses on the development of dental plaque, gingivitis, and discolouration. A randomized, investigator‐blind, placebo‐controlled, 3‐week experimental gingivitis study. J Clin Periodontol. 2006;33(8):561–567. doi: 10.1111/j.1600-051X.2006.00946.x 16899099

[37] Mesa F, Magán‐Fernández A, Muñoz R, et al. Catecholamine metabolites in urine, as chronic stress biomarkers, are associated with higher risk of chronic periodontitis in adults. J Periodontol. 2014;85(12):1755–1762. doi: 10.1902/jop.2014.140209 24965061

[38] Papapanou PN, Neiderud A, Papadimitriou A, et al. “Checkerboard” assessments of periodontal microbiota and serum antibody responses: a case‐control study. J Periodontol. 2000;71(6):885–897. doi: 10.1902/jop.2000.71.6.885 10914791

[39] Saxton CA, Lane RM, Van der Ouderaa F. The effects of a dentifrice containing a zinc salt and a non‐cationic antimicrobial agent on plaque and gingivitis. J Clin Periodontol. 1987;14(3):144–148. doi: 10.1111/j.1600-051X.1987.tb00957.x 3470319

[40] Sreenivasan P, Gaffar A. Antiplaque biocides and bacterial resistance: a review. J Clin Periodontol. 2002;29(11):965–974. doi: 10.1034/j.1600-051X.2002.291101.x 12472989

[41] Teles RP, Teles FRF. Antimicrobial agents used in the control of periodontal biofilms: effective adjuncts to mechanical plaque control? Braz Oral Res. 2009;23(suppl 1):39–48. doi: 10.1590/S1806-83242009000500007 19838557

[42] Zimmermann A, Flores‐de‐Jacoby L, Pan P, et al. Gingivitis, plaque accumulation and plaque composition under long‐term use of Meridol®. J Clin Periodontol. 1993;20(5):346–351. doi: 10.1111/j.1600-051X.1993.tb00371.x 8501274

[43] Hajishengallis G, Lamont RJ. Polymicrobial communities in periodontal disease: their quasi‐organismal nature and dialogue with the host. Periodontol 2000. 2021;86(1):210–230. doi: 10.1111/prd.12371 33690950 PMC8957750

[44] Darveau RP, Hajishengallis G, Curtis MA. Porphyromonas gingivalis as a potential community activist for disease. J Dent Res. 2012;91(9):816–820. doi: 10.1177/0022034512453589 22772362 PMC3420389

[45] Takeshita T, Yasui M, Shibata Y, et al. Dental plaque development on a hydroxyapatite disk in young adults observed by using a barcoded pyrosequencing approach. Sci Rep. 2015;5(1):1–9. doi: 10.1038/srep08136 PMC4311255 25633431

[46] Palmer RJ Jr, Shah N, Valm A, et al. Interbacterial adhesion networks within early oral biofilms of single human hosts. Appl Environ Microbiol. 2017;83(11):407. doi: 10.1128/AEM.00407-17 PMC5440702 28341674

[47] Sakamoto M, Li D, Shibata Y, et al. Porphyromonas pasteri sp. nov., isolated from human saliva. Int J Syst Evol Microbiol. 2015;65(8):2511–2515. doi: 10.1099/ijs.0.000294 25933621

[48] Yasunaga H, Takeshita T, Shibata Y, et al. Exploration of bacterial species associated with the salivary microbiome of individuals with a low susceptibility to dental caries. Clin Oral Investig. 2017;21(8):2399–2406. doi: 10.1007/s00784-016-2035-5 28013437

[49] Han YW, Ikegami A, Rajanna C, et al. Identification and characterization of a novel adhesin unique to oral fusobacteria. J Bacteriol. 2005;187(15):5330–5340. doi: 10.1128/JB.187.15.5330-5340.2005 16030227 PMC1196005

[50] Mayanagi G, Sato T, Shimauchi H, et al. Detection frequency of periodontitis‐associated bacteria by polymerase chain reaction in subgingival and supragingival plaque of periodontitis and healthy subjects. Oral Microbiol Immunol. 2004;19(6):379–385. doi: 10.1111/j.1399-302x.2004.00172.x 15491463

[51] Kook J, Park S, Lim YK, et al. Genome-based reclassification of Fusobacterium nucleatum subspecies at the species level. Curr Microbiol. 2017;74(10):1137–1147. doi: 10.1007/s00284-017-1296-9 28687946

[52] Chen T, Yu W, Izard J, et al. The human oral microbiome database: a web accessible resource for investigating oral microbe taxonomic and genomic information. Database. 2010;2010:baq013. doi: 10.1093/database/baq013 20624719 PMC2911848

[53] Belstrøm D, Paster BJ, Fiehn N, et al. Salivary bacterial fingerprints of established oral disease revealed by the human oral microbe identification using next generation sequencing (HOMI NGS) technique. J Oral Microbiol. 2016;8(1):30170. doi: 10.3402/jom.v8.30170 26782357 PMC4717152

[54] Cabral DJ, Wurster JI, Flokas ME, et al. The salivary microbiome is consistent between subjects and resistant to impacts of short-term hospitalization. Sci Rep. 2017;7(1):11040. doi: 10.1038/s41598-017-11427-2 28887570 PMC5591268

[55] Hiergeist A, Ruelle J, Emler S, et al. Reliability of species detection in 16S microbiome analysis: comparison of five widely used pipelines and recommendations for a more standardized approach. PLoS One. 2023;18(2):e0280870. doi: 10.1371/journal.pone.0280870 36795699 PMC9934417

[56] Teng F, Darveekaran Nair SS, Zhu P, et al. Impact of DNA extraction method and targeted 16S-rRNA hypervariable region on oral microbiota profiling. Sci Rep. 2018;8(1):16321. doi: 10.1038/s41598-018-34294-x 30397210 PMC6218491

[57] Verma D, Garg PK, Dubey AK. Insights into the human oral microbiome. Arch Microbiol. 2018;200(4):525–540. doi: 10.1007/s00203-018-1505-3 29572583

[58] Kawai T, Ohshima T, Shin R, et al. Determination of the antibacterial constituents produced by lactobacilli against a periodontal pathogen: Sodium lactate and a low molecular weight substance. J Prob Health. 2016;4(1359):1–7.

[59] Khalaf H, Nakka SS, Sandén C, et al. Antibacterial effects of Lactobacillus and bacteriocin PLNC8 αβ on the periodontal pathogen Porphyromonas gingivalis. BMC Microbiol. 2016;16(1):1–11. doi: 10.1186/s12866-016-0810-8 27538539 PMC4990846

[60] Pangsomboon K, Kaewnopparat S, Pitakpornpreecha T, et al. Antibacterial activity of a bacteriocin from Lactobacillus paracasei HL32 against Porphyromonas gingivalis. Arch Oral Biol. 2006;51(9):784–793. doi: 10.1016/j.archoralbio.2006.03.008 16870131

[61] Giordani B, Parolin C, Vitali B. Lactobacilli as anti-biofilm strategy in oral infectious diseases: a mini-review. Front Med Technol. 2021;3:769172. doi: 10.3389/fmedt.2021.769172 35047965 PMC8757881

[62] Sulijaya B, Yamada‐Hara M, Yokoji‐Takeuchi M, et al. Antimicrobial function of the polyunsaturated fatty acid KetoC in an experimental model of periodontitis. J Periodontol. 2019;90(12):1470–1480. doi: 10.1002/JPER.19-0130 31343074

[63] Zheng J, Wittouck S, Salvetti E, et al. A taxonomic note on the genus Lactobacillus: description of 23 novel genera, emended description of the genus lactobacillus beijerinck 1901, and union of lactobacillaceae and leuconostocaceae. Int J Syst Evol Microbiol. 2020;70(4):2782–2858. doi: 10.1099/ijsem.0.004107 32293557

[64] Chen Y, Hsieh P, Ho H, et al. Antibacterial activity of viable and heat‐killed probiotic strains against oral pathogens. Lett Appl Microbiol. 2020;70(4):310–317. doi: 10.1111/lam.13275 31955445

[65] Stensson M, Koch G, Coric S, et al. Oral administration of Lactobacillus reuteri during the first year of life reduces caries prevalence in the primary dentition at 9 years of age. Caries Res. 2014;48(2):111–117. doi: 10.1159/000354412 24296746

