# Supplementary material for: Screening of heat-killed lactic acid bacteria based on inhibitory activity against oral bacteria and effects of oral administration of heat-killed Ligilactobacillus salivarius CP3365 on periodontal health in healthy participants: a double-blinded, randomized, placebo-controlled trial
Source: J Oral Microbiol. 2023 Aug 28;15(1):2250649. doi: 10.1080/20002297.2023.2250649 (PMC10464545; doi:10.1080/20002297.2023.2250649)
Supplement: Supplemental Material [file ZJOM_A_2250649_SM9173.docx]

Supplementary Material

**Screening of heat-killed lactic acid bacteria based on inhibitory activity against oral bacteria and effects of oral administration of *Ligilactobacillus salivarius* CP3365 on oral health in healthy participants: A double-blinded, randomized, placebo-controlled trial**

Supplementary Figures and Tables

Supplementary Figures

**Supplementary Figure 1.** Rarefaction curves for an average number of observed features in each sample.

Supplementary Tables

Supplementary Table 1. Differences in the relative abundance of supra-gingival plaque microbiota at species level

| Taxonomic information | Groups | 0 weeks | | | | Detecton rate (%)^c^ | 8 weeks | | | | | Detection rate (%)^c^ | Δ8 weeks (8 w-0 w) | | | |
| --- | --- | --- | --- | --- | --- | --- | --- | --- | --- | --- | --- | --- | --- | --- | --- | --- |
| Species level |  | Mean |  | SE | *p*-value^a^ |  | Mean |  | SE | *p*-value^a^ | *p*-value^b^ |  | Mean |  | SE | p-value^a^ |
| *Fusobacterium* sp. | Placebo | 5.64 | ± | 0.61 | 0.17 | 100.0 | 4.28 | ± | 0.43 | 0.09 | 0.01 | 100.0 | -1.34 | ± | 0.46 | 0.96 |
|  | CP3365 | 6.52 | ± | 0.55 |  | 100.0 | 5.13 | ± | 0.52 |  | <0.01 | 100.0 | -1.40 | ± | 0.44 |  |
| *Fusobacterium nucleatum* subsp. *animalis* | Placebo | 0.97 | ± | 0.58 | 1.00 | 12.0 | 1.97 | ± | 0.65 | 1.00 | 0.11 | 12.0 | 1.00 | ± | 0.47 | 0.20 |
|  | CP3365 | 1.00 | ± | 1.00 |  | 8.0 | 1.55 | ± | 0.25 |  | 0.66 | 8.0 | 0.50 | ± | 1.30 |  |
| *Fusobacterium nucleatum* subsp. *vincentii* | Placebo | 0.48 | ± | 0.30 | 0.56 | 8.0 | 0.78 | ± | 0.32 | 0.91 | 0.42 | 12.0 | 0.30 | ± | 0.61 | 0.74 |
|  | CP3365 | 0.70 | ± | 0.31 |  | 12.0 | 0.63 | ± | 0.40 |  | 1.00 | 8.0 | -0.08 | ± | 0.69 |  |
| *Fusobacterium periodonticum* | Placebo | 1.97 | ± | 0.29 | 0.93 | 68.0 | 1.54 | ± | 0.25 | 0.93 | 0.23 | 64.0 | -0.43 | ± | 0.34 | 0.82 |
|  | CP3365 | 2.04 | ± | 0.30 |  | 64.0 | 1.68 | ± | 0.36 |  | 0.29 | 52.0 | -0.36 | ± | 0.43 |  |
| *Fusobacterium* sp. HMT 203 | Placebo | 1.53 | ± | 0.28 | 0.03 | 16.0 | 0.45 | ± | 0.45 | 1.00 | 0.07 | 16.0 | -1.08 | ± | 0.33 | 0.14 |
|  | CP3365 | 0.25 | ± | 0.10 |  | 16.0 | 0.40 | ± | 0.24 |  | 0.72 | 16.0 | 0.15 | ± | 0.34 |  |
| *Fusobacterium* sp. HMT 248 | Placebo |  |  |  | NT | ND | 3.10 |  |  | 0.50 | NT | 4.0 |  |  | . | NT |
|  | CP3365 | 0.63 | ± | 0.41 |  | 12.0 | 0.17 | ± | 0.17 |  | 0.41 | 12.0 | -0.47 | ± | 0.55 |  |
| *Haemophilus* sp. | Placebo | 0.41 | ± | 0.12 | 0.14 | 40.0 | 0.96 | ± | 0.48 | 0.51 | 0.36 | 36.0 | 0.55 | ± | 0.51 | 0.23 |
|  | CP3365 | 1.07 | ± | 0.38 |  | 36.0 | 0.48 | ± | 0.20 |  | 0.24 | 20.0 | -0.66 | ± | 0.48 |  |
| *Haemophilus parainfluenzae* | Placebo | 2.56 | ± | 0.41 | 0.32 | 84.0 | 2.59 | ± | 0.47 | 0.20 | 0.85 | 80.0 | 0.02 | ± | 0.37 | 0.57 |
|  | CP3365 | 2.96 | ± | 0.36 |  | 84.0 | 3.15 | ± | 0.36 |  | 0.58 | 84.0 | 0.20 | ± | 0.43 |  |
| *Haemophilus pittmaniae* | Placebo |  |  |  | NT | ND |  |  |  |  | NT | ND |  |  |  | NT |
|  | CP3365 | 0.80 |  |  |  | 4.0 | 2.00 |  |  |  | 0.32 | 4.0 | 1.20 |  |  |  |
| *Haemophilus* sp. HMT 036 | Placebo | 0.77 | ± | 0.24 | 0.31 | 32.0 | 1.65 | ± | 0.67 | 0.74 | 0.55 | 36.0 | 0.87 | ± | 0.76 | 0.66 |
|  | CP3365 | 1.43 | ± | 0.42 |  | 28.0 | 1.16 | ± | 0.30 |  | 0.51 | 32.0 | -0.28 | ± | 0.51 |  |
| *Haemophilus* sp. HMT 908 | Placebo |  |  |  | NT | ND |  |  |  | NT | NT | ND |  |  |  | NT |
|  | CP3365 |  |  |  |  | ND | 0.10 |  |  |  | 0.32 | 4.0 | 0.10 |  |  |  |
| *Neisseria* sp. | Placebo | 4.63 | ± | 0.90 | 0.51 | 76.0 | 4.78 | ± | 0.84 | 0.69 | 0.67 | 92.0 | 0.13 | ± | 0.67 | 0.04 |
|  | CP3365 | 5.81 | ± | 1.04 |  | 100.0 | 4.47 | ± | 0.85 |  | 0.02 | 72.0 | -1.34 | ± | 0.75 |  |
| *Neisseria bacilliformis* | Placebo | 0.10 | ± | 0.10 | 0.80 | 4.0 | 1.05 | ± | 0.36 | 0.53 | 0.07 | 16.0 | 0.95 | ± | 0.26 | 0.45 |
|  | CP3365 |  |  |  |  | ND | 1.70 | ± | 0.30 |  | 0.18 | 8.0 |  |  |  |  |
| *Neisseria elongata* | Placebo | 0.59 | ± | 0.21 | 0.18 | 20.0 | 0.93 | ± | 0.34 | 0.64 | 0.48 | 28.0 | 0.34 | ± | 0.34 | 0.35 |
|  | CP3365 | 1.41 | ± | 0.37 |  | 40.0 | 0.99 | ± | 0.24 |  | 0.31 | 44.0 | -0.47 | ± | 0.54 |  |
| *Neisseria oralis* | Placebo | 0.86 | ± | 0.48 | 0.79 | 24.0 | 0.89 | ± | 0.25 | 0.90 | 0.61 | 28.0 | 0.04 | ± | 0.49 | 0.53 |
|  | CP3365 | 0.71 | ± | 0.20 |  | 32.0 | 0.95 | ± | 0.24 |  | 0.51 | 40.0 | 0.26 | ± | 0.32 |  |
| *Neisseria* sp. HMT 018 | Placebo | 1.35 | ± | 1.22 | 0.40 | 8.0 | 1.15 | ± | 0.18 | 0.63 | 0.72 | 16.0 | -0.20 | ± | 1.32 | 0.07 |
|  | CP3365 | 1.07 | ± | 0.42 |  | 12.0 | 0.60 | ± | 0.60 |  | 0.11 | 4.0 | -0.47 | ± | 0.19 |  |
| *Porphyromonas* sp. | Placebo | 0.72 | ± | 0.26 | 0.09 | 44.0 | 0.74 | ± | 0.22 | 0.40 | 0.65 | 40.0 | 0.01 | ± | 0.27 | 1.00 |
|  | CP3365 | 1.12 | ± | 0.25 |  | 56.0 | 1.04 | ± | 0.24 |  | 0.98 | 48.0 | -0.08 | ± | 0.32 |  |
| *Porphyromonas catoniae* | Placebo | 2.40 |  |  | 0.40 | 4.0 | 0.60 | ± |  | 1.00 | 0.32 | 4.0 | -1.80 | ± | . | 0.64 |
|  | CP3365 | 0.40 | ± | 0.31 |  | 8.0 | 0.48 | ± | 0.29 |  | 1.00 | 8.0 | 0.08 | ± | 0.55 |  |
| *Porphyromonas endodontalis* | Placebo | 0.65 | ± | 0.20 | 0.58 | 32.0 | 0.54 | ± | 0.19 | 0.46 | 0.80 | 32.0 | -0.12 | ± | 0.29 | 0.18 |
|  | CP3365 | 0.69 | ± | 0.18 |  | 44.0 | 0.37 | ± | 0.13 |  | 0.04 | 32.0 | -0.35 | ± | 0.13 |  |
| *Porphyromonas gingivalis* | Placebo | 0.20 | ± | 0.20 | 1.00 | 4.0 | 0.20 | ± | 0.20 | 1.00 | 1.00 | 4.0 | 0.00 | ± | 0.40 | 0.97 |
|  | CP3365 | 0.57 | ± | 0.47 |  | 8.0 | 0.20 | ± | 0.10 |  | 0.41 | 12.0 | -0.45 | ± | 0.55 |  |
| *Porphyromonas pasteri* | Placebo | 3.63 | ± | 0.66 | 0.95 | 84.0 | 4.71 | ± | 1.03 | 0.38 | 0.13 | 100.0 | 1.11 | ± | 0.56 | 0.06 |
|  | CP3365 | 3.74 | ± | 0.75 |  | 88.0 | 3.09 | ± | 0.48 |  | 0.23 | 96.0 | -0.66 | ± | 0.51 |  |
| *Porphyromonas* sp. HMT 278 | Placebo | 1.15 | ± | 0.41 | 0.91 | 36.0 | 0.65 | ± | 0.30 | 0.72 | 0.06 | 32.0 | -0.51 | ± | 0.24 | 0.37 |
|  | CP3365 | 0.90 | ± | 0.23 |  | 52.0 | 0.45 | ± | 0.16 |  | 0.05 | 32.0 | -0.45 | ± | 0.23 |  |
| *Porphyromonas* sp. HMT 930 | Placebo | 0.95 | ± | 0.32 | 0.84 | 20.0 | 0.15 | ± | 0.10 | 0.73 | 0.06 | 8.0 | -0.80 | ± | 0.31 | 0.76 |
|  | CP3365 | 0.79 | ± | 0.22 |  | 24.0 | 0.30 | ± | 0.18 |  | 0.06 | 12.0 | -0.49 | ± | 0.20 |  |
| *Porphyromonas uenonis* | Placebo | 0.00 |  |  | NT | 0.0 | 0.20 |  |  |  | 0.32 | 4.0 | 0.20 |  |  | NT |
|  | CP3365 |  |  |  |  | 0.0 |  |  |  |  |  | 0.0 |  |  |  |  |

^a^The parameters between placebo and CP3365 were evaluated using Wilcoxon rank-sum test. ^b^The parameters between 0 weeks and 8 weeks were evaluated using Wilcoxon signed-rank test. ^c^The number of samples in each group was 25. ND; Not detected, NT; Not tested.

Supplementary Table 2. Differences in the relative abundance of salivary microbiota at species level

| Taxonomic information | Groups | 0 weeks | | | | Detection rate (%) | 8 weeks | | | | | Detection rate (%) | Δ8 weeks (8 w-0 w) | | | |
| --- | --- | --- | --- | --- | --- | --- | --- | --- | --- | --- | --- | --- | --- | --- | --- | --- |
| Species level |  | Mean |  | SE | p-value^a^ |  | Mean |  | SE | p-value^a^ | p-value^b^ |  | Mean |  | SE | p-value^a^ |
| *Fusobacterium* sp. | Placebo | 1.03 | ± | 0.22 | 0.09 | 81.5 | 1.40 | ± | 0.20 | 0.40 | 0.03 | 96.3 | 0.39 | ± | 0.19 | 0.34 |
|  | CP3365 | 1.46 | ± | 0.26 |  | 80.8 | 1.60 | ± | 0.23 |  | 0.11 | 84.6 | 0.16 | ± | 0.21 |  |
| *Fusobacterium necrophorum* | Placebo | 0.30 | ± | 0.30 | NT | 3.7 | 0.30 | ± | 0.28 | 0.67 | 1.00 | 7.4 | 0.10 |  | - | NT |
|  | CP3365 |  |  |  |  | ND | 0.10 | ± | 0.00 |  | 0.16 | 7.7 |  |  |  |  |
| *Fusobacterium nucleatum* subsp. *vincentii* | Placebo | 0.53 | ± | 0.26 | 0.86 | 18.5 | 0.74 | ± | 0.30 | 0.66 | 0.50 | 18.5 | 0.21 | ± | 0.30 | 0.55 |
|  | CP3365 | 0.43 | ± | 0.15 |  | 23.1 | 0.82 | ± | 0.16 |  | 0.09 | 42.3 | 0.49 | ± | 0.27 |  |
| *Fusobacterium periodonticum* | Placebo | 7.71 | ± | 0.79 | 0.09 | 96.3 | 7.43 | ± | 0.83 | 0.03 | 0.77 | 100.0 | -0.29 | ± | 0.64 | 0.51 |
|  | CP3365 | 5.85 | ± | 0.69 |  | 100.0 | 4.95 | ± | 0.67 |  | 0.17 | 96.2 | -0.88 | ± | 0.53 |  |
| *Fusobacterium* sp. HMT 203 | Placebo |  |  |  | NT | ND |  |  |  | NT | NT | ND |  |  |  | NT |
|  | CP3365 | 0.65 | ± | 0.65 |  | 7.7 | 0.90 | ± | 0.10 |  | 0.66 | 7.7 | 0.25 | ± | 0.55 |  |
| *Fusobacterium* sp. HMT 248 | Placebo | 0.32 | ± | 0.16 | 0.79 | 18.5 | 0.56 | ± | 0.39 | 0.79 | 0.79 | 18.5 | 0.24 | ± | 0.48 | 0.56 |
|  | CP3365 | 0.23 | ± | 0.15 |  | 23.1 | 0.38 | ± | 0.14 |  | 0.59 | 23.1 | 0.16 | ± | 0.29 |  |
| *Haemophilus* sp. | Placebo | 0.27 | ± | 0.09 | 0.23 | 44.4 | 0.40 | ± | 0.07 | 0.49 | 0.06 | 66.7 | 0.14 | ± | 0.09 | 0.20 |
|  | CP3365 | 0.17 | ± | 0.10 |  | 23.1 | 0.56 | ± | 0.14 |  | 0.00 | 53.8 | 0.38 | ± | 0.10 |  |
| *Haemophilus parainfluenzae* | Placebo | 3.91 | ± | 0.37 | 0.29 | 96.3 | 4.24 | ± | 0.27 | 0.03 | 0.30 | 100.0 | 0.33 | ± | 0.35 | 0.31 |
|  | CP3365 | 3.37 | ± | 0.23 |  | 100.0 | 3.38 | ± | 0.26 |  | 0.98 | 100.0 | 0.00 | ± | 0.22 |  |
| *Haemophilus paraphrohaemolyticus* | Placebo |  |  |  | NT | ND | 0.40 |  |  | NT | 0.32 | 3.7 | 0.40 |  |  | NT |
|  | CP3365 |  |  |  |  | ND |  |  |  |  | NT | ND |  |  |  |  |
| *Haemophilus pittmaniae* | Placebo | 0.40 | ± | 0.19 | 0.35 | 11.1 | 1.08 | ± | 0.70 | 0.48 | 0.59 | 11.1 | 0.70 | ± | 0.85 | 0.21 |
|  | CP3365 | 0.92 | ± | 0.40 |  | 19.2 | 0.38 | ± | 0.21 |  | 0.25 | 11.5 | -0.53 | ± | 0.35 |  |
| *Haemophilus* sp. HMT 036 | Placebo | 0.12 | ± | 0.04 | 0.62 | 25.9 | 0.49 | ± | 0.15 | 0.25 | 0.03 | 40.7 | 0.40 | ± | 0.18 | 0.52 |
|  | CP3365 | 0.15 | ± | 0.04 |  | 38.5 | 0.27 | ± | 0.05 |  | 0.07 | 46.2 | 0.14 | ± | 0.07 |  |
| *Haemophilus* sp. HMT 908 | Placebo | 0.25 | ± | 0.19 | 0.80 | 7.4 | 0.20 | ± | 0.07 | 0.40 | 0.85 | 11.1 | -0.05 | ± | 0.18 | 0.64 |
|  | CP3365 | 0.00 |  |  |  | ND | 0.50 |  |  |  | 0.32 | 3.8 | 0.50 |  |  |  |
| *Haemophilus sputorum* | Placebo | 0.34 | ± | 0.08 | 0.05 | 25.9 | 0.23 | ± | 0.08 | 0.60 | 0.21 | 22.2 | -0.14 | ± | 0.11 | 0.20 |
|  | CP3365 | 0.05 | ± | 0.05 |  | 3.8 | 0.28 | ± | 0.09 |  | 0.14 | 11.5 | 0.23 | ± | 0.14 |  |
| *Neisseria* sp. | Placebo | 4.03 | ± | 0.39 | 0.31 | 96.3 | 4.14 | ± | 0.36 | 0.70 | 0.65 | 100.0 | 0.12 | ± | 0.29 | 0.28 |
|  | CP3365 | 5.59 | ± | 0.80 |  | 100.0 | 4.64 | ± | 0.44 |  | 0.24 | 100.0 | -1.03 | ± | 0.70 |  |
| *Neisseria bacilliformis* | Placebo |  |  |  | NT | ND | 0.57 | ± | 0.15 | 0.50 | NT | 11.1 |  |  |  | NT |
|  | CP3365 |  |  |  |  | ND | 0.30 | ± |  |  | NT | 3.8 |  |  |  |  |
| *Neisseria elongata* | Placebo | 0.38 | ± | 0.09 | 0.86 | 59.3 | 0.42 | ± | 0.09 | 0.77 | 0.86 | 63.0 | 0.03 | ± | 0.12 | 0.76 |
|  | CP3365 | 0.42 | ± | 0.12 |  | 57.7 | 0.42 | ± | 0.09 |  | 0.98 | 65.4 | -0.01 | ± | 0.11 |  |
| *Neisseria oralis* | Placebo | 0.01 | ± | 0.01 | 0.08 | 3.7 | 0.30 | ± | 0.08 | 0.14 | <0.01 | 37.0 | 0.29 | ± | 0.08 | 0.40 |
|  | CP3365 | 0.19 | ± | 0.07 |  | 23.1 | 0.55 | ± | 0.15 |  | <0.01 | 46.2 | 0.37 | ± | 0.09 |  |
| *Neisseria* sp. HMT 018 | Placebo | 0.24 | ± | 0.16 | 1.00 | 7.4 | 0.42 | ± | 0.25 | 0.39 | 0.69 | 11.1 | 0.18 | ± | 0.37 | 0.50 |
|  | CP3365 | 0.13 | ± | 0.07 |  | 7.7 | 0.07 | ± | 0.07 |  | 0.56 | 3.8 | -0.07 | ± | 0.13 |  |
| *Neisseria* sp. HMT 499 | Placebo | 0.20 |  |  | NT | 3.7 |  |  |  | NT | ND | ND |  |  |  | NT |
|  | CP3365 |  |  |  |  | ND |  |  |  |  | ND | ND |  |  |  |  |
| *Porphyromonas* sp. | Placebo | 0.62 | ± | 0.13 | 0.49 | 74.1 | 0.51 | ± | 0.11 | 0.96 | 0.38 | 66.7 | -0.12 | ± | 0.12 | 0.78 |
|  | CP3365 | 0.73 | ± | 0.13 |  | 80.8 | 0.54 | ± | 0.12 |  | 0.22 | 61.5 | -0.21 | ± | 0.15 |  |
| *Porphyromonas catoniae* | Placebo | 0.20 | ± | 0.20 | 1.00 | 3.7 | 0.15 | ± | 0.15 | 0.19 | 0.66 | 3.7 | -0.05 | ± | 0.35 | 0.64 |
|  | CP3365 | 0.30 | ± | 0.10 |  | 15.4 | 0.40 | ± | 0.07 |  | 0.41 | 19.2 | 0.17 | ± | 0.20 |  |
| *Porphyromonas endodontalis* | Placebo | 0.33 | ± | 0.06 | 0.89 | 63.0 | 0.52 | ± | 0.16 | 0.56 | 0.34 | 74.1 | 0.22 | ± | 0.17 | 0.37 |
|  | CP3365 | 0.34 | ± | 0.06 |  | 73.1 | 0.34 | ± | 0.07 |  | 0.92 | 61.5 | -0.01 | ± | 0.09 |  |
| *Porphyromonas gingivalis* | Placebo | 0.27 | ± | 0.04 | 0.74 | 25.9 | 0.26 | ± | 0.09 | 0.54 | 0.49 | 22.2 | -0.04 | ± | 0.14 | 0.09 |
|  | CP3365 | 0.27 | ± | 0.07 |  | 30.8 | 0.54 | ± | 0.25 |  | 0.23 | 38.5 | 0.39 | ± | 0.26 |  |
| *Porphyromonas pasteri* | Placebo | 6.64 | ± | 0.48 | 0.14 | 100.0 | 4.87 | ± | 0.41 | 0.14 | <0.01 | 100.0 | -1.78 | ± | 0.38 | 0.44 |
|  | CP3365 | 5.37 | ± | 0.46 |  | 100.0 | 3.83 | ± | 0.38 |  | <0.01 | 100.0 | -1.60 | ± | 0.35 |  |
| *Porphyromonas* sp. HMT 278 | Placebo | 0.36 | ± | 0.07 | 0.31 | 70.4 | 0.40 | ± | 0.09 | 0.03 | 0.82 | 63.0 | 0.04 | ± | 0.11 | 0.98 |
|  | CP3365 | 0.90 | ± | 0.26 |  | 46.2 | 0.77 | ± | 0.15 |  | 0.90 | 61.5 | -0.13 | ± | 0.19 |  |
| *Porphyromonas* sp. HMT 285 | Placebo | 0.10 |  |  | 1.00 | 3.7 |  |  |  | NT | NT | ND |  |  |  | NT |
|  | CP3365 | 0.20 |  |  |  | 3.8 |  |  |  |  | NT | ND |  |  |  |  |
| *Porphyromonas* sp. HMT 930 | Placebo | 0.53 | ± | 0.17 | 0.75 | 37.0 | 0.42 | ± | 0.10 | 0.40 | 0.60 | 44.4 | -0.11 | ± | 0.22 | 0.33 |
|  | CP3365 | 0.33 | ± | 0.09 |  | 34.6 | 0.60 | ± | 0.16 |  | 0.08 | 38.5 | 0.28 | ± | 0.15 |  |
| *Porphyromonas uenonis* | Placebo | 0.03 | ± | 0.03 | NT | 3.7 | 0.17 | ± | 0.03 | NT | 0.10 | 11.1 | 0.15 | ± | 0.05 | NT |
|  | CP3365 |  |  |  | NT | ND |  |  |  |  | NT | ND |  |  |  |  |

^a^The parameters between placebo and CP3365 were evaluated using Wilcoxon rank-sum test. ^b^The parameters between 0 weeks and 8 weeks were evaluated using Wilcoxon signed-rank test. ^c^The number of samples in the placebo group was 27, and that in the CP3365 group was 26. ND; Not detected, NT; Not tested.
